# Protein Tyrosine Phosphatase-1B Inhibition Disrupts IL13Rα2-Promoted Invasion and Metastasis in Cancer Cells

**DOI:** 10.3390/cancers12020500

**Published:** 2020-02-21

**Authors:** Rubén A. Bartolomé, Ángela Martín-Regalado, Marta Jaén, Markella Zannikou, Peng Zhang, Vivian de los Ríos, Irina V. Balyasnikova, J. Ignacio Casal

**Affiliations:** 1 Department of Molecular Biomedicine, Centro de Investigaciones Biológicas, CSIC, Ramiro de Maeztu 9, 28039 Madrid, Spain; rubenabc@cib.csic.es (R.A.B.); martajaen@cib.csic.es (M.J.); 2 Department of Neurological Surgery, Feinberg School of Medicine, Northwestern University, Chicago, IL 60611, USA; markella.zannikou@northwestern.edu (M.Z.); peng@northwestern.edu (P.Z.); irinabal@northwestern.edu (I.V.B.); 3 Proteomics Core Facility, Centro de Investigaciones Biológicas (CSIC), 28001 Madrid, Spain; vrios@cib.csic.es

**Keywords:** colorectal cancer, EGFR, glioblastoma, IL13Rα2, metastasis, ovarian cancer, PTP1B, Src

## Abstract

*Background:* Interleukin 13 receptor alpha 2 subunit (IL13Rα2) is overexpressed in glioblastoma (GBM), metastatic colorectal cancer (CRC) and ovarian cancer (OC). Here, we investigated the IL13Rα2 interactome searching for novel targets in cancer invasion and metastasis. *Methods:* The interactome of IL13Rα2 was determined in GBM by using a proteomic analysis and then validated in CRC and OC. Cell signaling was investigated using siRNA interference, protein tyrosine phosphatase-1B (PTP1B) inhibitors and Western blot analysis. Animal models of GBM and metastatic CRC were used for testing PTP1B inhibitors. *Results:* PTP1B was identified and validated as a mediator of IL13Rα2 signaling. An in silico analysis revealed that PTP1B overexpression is associated with lower overall survival of patients in the three types of cancer. PTP1B silencing or treatment with Claramine, a PTP1B inhibitor, caused a significant decrease in IL-13-mediated adhesion, migration and invasion of IL13Rα2-expressing cancer cells by inhibiting the dephosphorylation of Src Tyr_530_ and consequently, the phosphorylation of Src Tyr_419_, AKT and ERK1/2. In addition, Claramine inhibited EGF-mediated activation of EGFR Tyr_1068._ In vivo treatment with Claramine caused a total inhibition of liver metastasis in mice inoculated with CRC cells and a significant increase in the survival of mice bearing intracranial GBM patient-derived xenografts. *Conclusions:* We have uncovered that IL13 signaling through IL13Rα2 requires PTP1B activity and therefore, PTP1B inhibition represents a promising therapeutic strategy in multiple types of cancer, including glioblastoma.

## 1. Introduction

IL13Rα2 has been classified as a cancer/testis-like tumor antigen encoded in chromosome X [1]. In normal tissues, IL13Rα2 is mainly expressed in the testis. However, IL13Rα2 is overexpressed in a variety of tumor types such as colorectal cancer (CRC), renal cell carcinoma, pancreatic, melanoma, head and neck, mesothelioma, ovarian cancer (OC) and glioblastoma (GBM), among others [2,3,4,5,6,7,8,9]. In CRC, a statistically significant association was observed between IL13Rα2 expression and tumor progression (T stage), with higher expression in T3 or T4 tumors as compared with T1 or T2 [10]. In ovarian cancer (OC), IL-13 was described to regulate cancer invasion and metastasis through IL13Rα2 [11]. In GBM, IL13Rα2 is upregulated following the expression of mutant EGFRvIII [12], being overexpressed in 58% of adult and 83% of pediatric brain tumors [13,14]. Furthermore, IL13Rα2 expression in GBM has been associated with an increased malignancy grade, an aggressive mesenchymal gene expression signature, and a poorer patient prognosis [15]. Despite advances in neurosurgery and adjuvant treatment, the median survival of patients with GBM is only about 21 months and no improvements in overall patient survival have been obtained in the last 50 years [16].

Due to the specific expression in cancer cells, the IL13Rα2 has been validated as target for cancer therapy in multiple studies and trough different approaches (see [17] for a review). Initially, the IL-13/IL-13Rα2 axis was demonstrated to mediate signaling through AP-1 transcriptional pathway in a number of human cancers [3,11,18,19]. The AP-1 pathway lies at the bottom of the Ras → Raf → MAPK cascade. In CRC cells, the binding of IL-13 to IL13Rα2 triggers STAT6-independent cellular pathways, promoting migration, invasion and survival of cancer cells through the scaffold protein FAM120A, which participates in the activation of FAK and the PI3K pathway [10,20]. Therefore, IL-13/IL-13Rα2 use the PI3K/AKT/mTOR and MAP kinases signaling that, in turn, induces the activation of the AP-1 complex to promote human cancer metastasis. However, the connection between IL13Rα2 and Src was unclear, as the IL13Rα2 cytoplasmic domain is relatively short for stable protein–protein interactions. Recently, we have reported that FAM120A may act as a bridge between IL13Rα2 and Src [20]. Furthermore, a candidate IL13Rα2 therapeutic peptide was able to inhibit IL-13 signaling capacity in both tumors, CRC and glioblastoma, by inhibiting the Src pathway [21].

Protein-tyrosine phosphatases (PTPs) work in a coordinated way to regulate tyrosine kinases phosphorylation and consequently, several fundamental physiological processes. Among them, PTP1B is a multifunctional protein associated to the glucose metabolism that plays a key role in multiple diseases like obesity, diabetes or cancer [22]. PTP1B suppression decreases blood glucose and insulin levels, increasing insulin sensitivity [23]. PTP1B KO mice are resistant to type II diabetes and obesity. In fact, patients with type II diabetes show a higher cancer risk and worse prognosis [24]. After some initial conflicting results, PTP1B has been shown to work more like an oncogene than a tumor suppressor. Increased PTP1B expression has been reported in colorectal [25,26], prostate [27], breast [28], ovarian [29] and gastric [30] cancers. In colorectal and gastric cancer, increased expression of PTP1B was associated to tumor progression and poor patient outcome [26,31]. PTP1B promotes proliferation and metastasis through the activation of Src/Ras/ERK and PI3K/AKT signaling pathways [32], in a similar way to IL-13. Src activation is regulated by PTP1B through the dephosphorylation of Tyr_530_ that interacts with the SH2 domain to suppress its kinase activity [33,34]. However, no association of PTP1B with IL13Rα2 signaling has been previously described.

A better understanding of the IL13/IL13Rα2 signaling pathway would facilitate the discovery of novel therapeutic candidates in those cancers characterized by high expression of IL13Rα2, such as colorectal, ovarian, or GBM. This study provides evidence for the first time that PTP1B mediates IL13Rα2 pro-tumorigenic activities in the three types of cancer and may provide a novel therapeutic target to inhibit IL13/IL13Rα2 signaling.

## 2. Results

### 2.1. PTP1B Associates with IL13Rα2 in Cancer Cells

**Figure 1:**
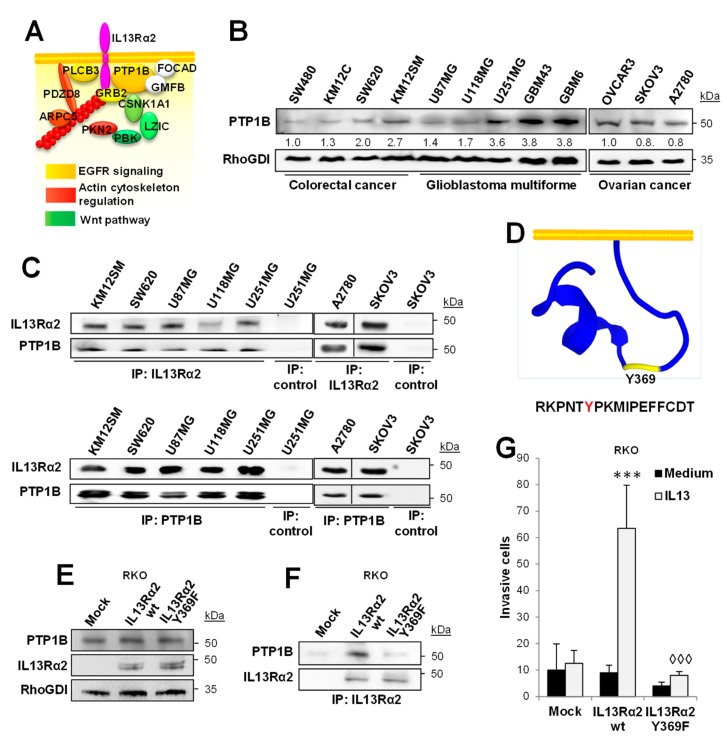
PTP1B associates with IL13Rα2. (**A**) Protein interaction network for IL13Rα2 in U251 cells was identified by mass spectrometry analysis. Protein position was tentatively assigned based on the existing literature. (**B**) Western blot analysis of the expression of PTP1B in human CRC, OC and GMB cell lines and PDXs (GBM43 and GBM6). (**C**) Western blot analysis of IL13Rα2 (top) and PTP1B (bottom) co-immunoprecipitated proteins in the indicated cell lines. (**D**) PEP-FOLD3 representation of IL13Rα2 cytoplasmic tail structure showing the Tyr_369_ location. Colorectal RKO cancer cells transfected with empty vectors (mock) or vectors encoding for wild type IL13Rα2 or Y369F mutated form were lysed and subjected to (**E**) Western blot analysis to verify the expression of PTP1B and IL13Rα2 forms and (**F**) IL13Rα2 immunoprecipitation for the detection of coimmunoprecipitated PTP1B. (**G**) The same RKO transfectants were subjected to invasion through Matrigel. Whereas IL-13 promoted cell invasion of IL13Rα2 WT cells (*** *p* < 0.001), the Tyr_369_ mutant significantly inhibited the invasion (◊◊◊ *p* < 0.001).

To characterize the protein interaction network of IL13Rα2, whole-cell lysates of the human GBM cell line U251 were immunoprecipitated and analyzed by mass spectrometry. Eleven proteins were found to be specifically associated with IL13Rα2 after removing those proteins immunoprecipitated with a control antibody or involved in non-specific protein-binding functions (i.e., chaperons and ribosomal proteins). A schematic representation of the interacting proteins and their tentative location is depicted in Figure 1A. Based on the number of identified peptides, the phosphatase PTP1B was selected as the most relevant IL13Rα2 interaction partner (Supplementary Table S1). PTP1B expression was analyzed in a panel of four IL13Rα2 positive CRC cell lines [10], three GBM cell lines, two GBM patient-derived xenografts (PDXs) and three OC cell lines (Figure 1B). Although all the cells expressed PTP1B, the highest level of PTP1B protein was found in the GBM PDXs. The association of PTP1B with IL13Rα2 was confirmed in the different cell lines by co-IP with either PTP1B or IL13Rα2-specific antibodies followed by Western blot detection (Figure 1C).

One of the motifs used by PTP1B for substrate recognition is [RK][AGST][LIV]XXpY [35], which resembles the sequence RKPNTY_369_ contained in the cytoplasmic tail of IL13Rα2. Therefore, we hypothesized that Tyr_369_ of the IL13Rα2 cytoplasmic tail could be the anchor point for PTP1B (Figure 1D). To assess this hypothesis, we prepared the mutant Tyr_369_Phe and transfected both, wild type and mutant IL13Rα2, in RKO CRC cells, which do not express IL13Rα2. The expression of wild-type and mutant IL13Rα2, as well as the endogenous expression of PTP1B in RKO cells, was verified by Western blot (Figure 1E). After IL13Rα2 IP, PTP1B was found to be exclusively associated with the wild-type IL13Rα2, but not with the mutant form Phe_369_ (Figure 1F). Moreover, RKO cells containing the mutant Tyr369Phe showed a clear inhibition of the invasive properties (Figure 1G). Taken together, these results support a role for the phosphorylated Tyr_369_ in the pro-invasive effects of IL13Rα2 through PTP1B binding.

In addition, we investigated whether knocking down PTP1B or IL13Rα2 might affect the expression and localization of each other. After the treatment with IL-13, cancer cells knocked down for PTP1B, showed an increase of IL13Rα2 on the cell surface (Supplementary Figure S1A) together with less protein degradation (Supplementary Figure S1B). In contrast, knocking down IL13Rα2 did not cause any effect on PTP1B expression (Supplementary Figure S1C). Therefore, PTP1B silencing reduces IL13Rα2 internalization and degradation in cancer cells.

### 2.2. PTP1B Overexpression Is Associated with a Lower Overall Survival of Patients

**Figure 2:**
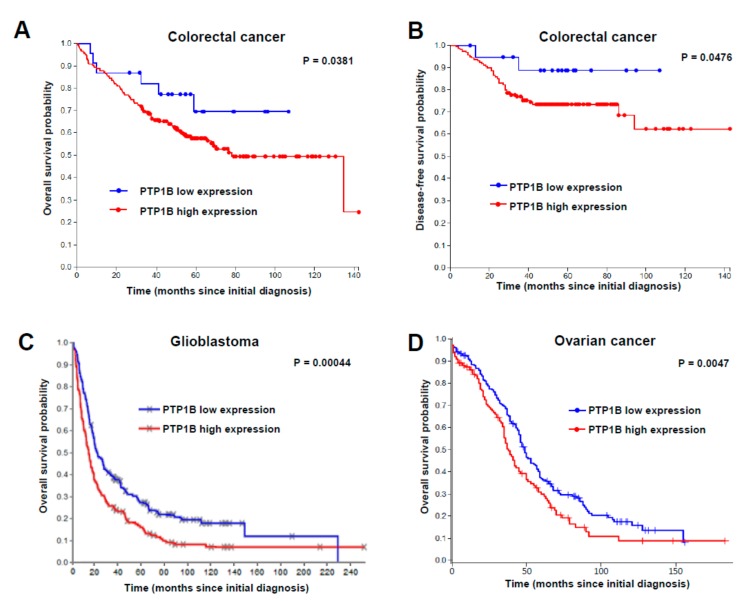
Prognostic value of PTP1B in cancer patients. Kaplan–Meier survival analysis in (**A**,**B**) colorectal cancer, (**C**) glioblastoma and (**D**) ovarian cancer patients, according to PTP1B mRNA expression. Significant associations of PTP1B expression with lower overall survival were found in the three types of cancer using the log-rank statistical method.

To study the clinical relevance of PTP1B, we carried out “in silico” studies of PTP1B expression. For human colorectal cancer, we performed an in silico analysis of the GSE17538 dataset. Although the z-score for PTP1B expression was not distributed in a Gaussian fashion, 90% of the tumor samples expressed significantly higher levels of PTP1B. Then, a significantly negative correlation was found between PTP1B expression levels and overall (Figure 2A) or disease-free survival (Figure 2B) for colorectal cancer patients. To investigate the relevance of PTP1B expression in glioma patients, we used the REMBRANDT data repository. Using the median as a threshold, we found a significantly reduced overall survival of GBM patients with high PTP1B expression (Figure 2C). PTP1B expression in OC was analyzed using the GEPIA2 database. The results indicate an association of high PTP1B expression with lower overall survival (Figure 2D). However, in silico analysis did not show a significant correlation between PTP1B and IL13Rα2 expression. Collectively, these results support an association between increased PTP1B expression and poorer patient outcome in the three types of cancer.

### 2.3. PTP1B Mediates IL13-Induced Cancer Cell Proliferation, Migration, Invasion and Survival

**Figure 3:**
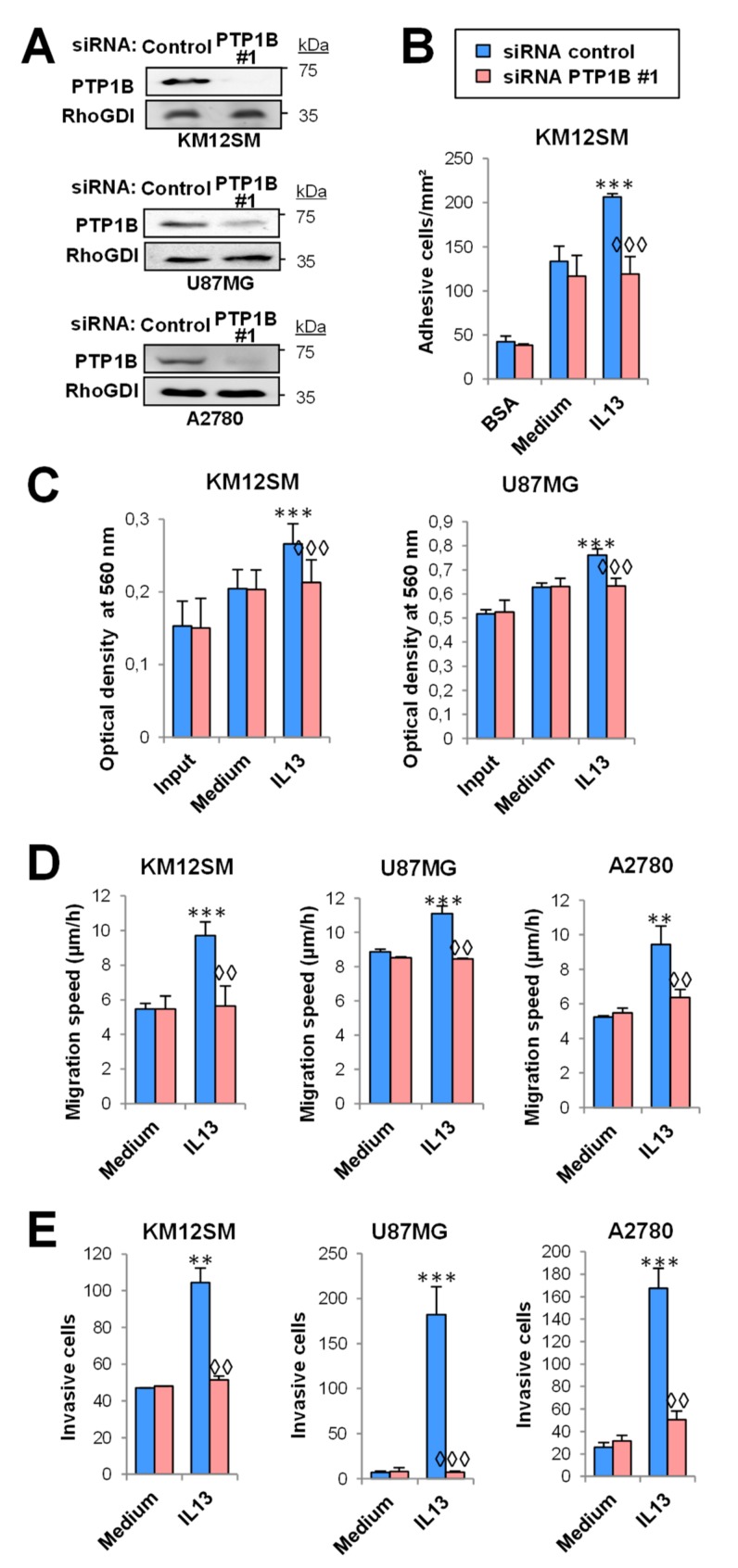
PTP1B mediates IL-13-triggered cell adhesion, migration, invasion and proliferation in cancer cells. U87MG, KM12SM, and A2780 cells were transfected with control or PTP1B siRNA #1. (**A**) PTP1B silencing was verified 48 h after transfection by Western blot. (**B**) Colorectal cancer transfectants were subjected to cell adhesion. (**C**) Glioblastoma and colorectal cancer transfectants were subjected to MTT. Then, the three cancer transfectants were subjected to (**D**) migration and (**E**) invasion assays. All assays were done in the presence or absence of IL-13. Cell adhesion/migration/invasion/optical density was significantly increased by addition of IL-13 (** *p* < 0.01; *** *p* < 0.001) and declined after PTP1B silencing (◊◊ *p* < 0.01; ◊◊◊ *p* < 0.001).

To investigate the activity of PTP1B in the pro-invasive and metastatic processes induced by IL-13 in cancer cells, we prepared KM12SM/SW620 CRC cells, U118/U87 GBM cells and A2780/SKOV3 OC cells PTP1B-silenced using two different siRNAs (Figure 3A, Supplementary Figure S2A). PTP1B-silenced and control cells were investigated for cell adhesion, proliferation, migration, and invasion in the presence or absence of IL-13. Experiments were performed in absence of serum, except proliferation (0.5% serum). After IL-13 addition, only CRC cells showed an apparent increase in cell adhesion that was inhibited by PTP1B silencing (Figure 3B, Supplementary Figure S2B). IL-13 effect on proliferation increase was restricted to CRC and GBM with no effect in OC cells and was inhibited after PTP1B silencing (Figure 3C, Supplementary Figure S2C). The IL-13-promoted migration and invasion were decreased by PTP1B silencing in all tested CRC, OC and GBM cell lines (Figure 3D,E, Supplementary Figure S2D,E). In addition, as PTP1B has been involved in the activation of the PI3K/AKT pathway, we tested the effect of IL-13 and PTP1B on cell survival. The presence of IL-13 improved the survival of cells subjected to oxidative stress. This survival increase was abolished after PTP1B silencing with two different siRNAs (Supplementary Figure S3). Collectively, these results support that IL13-induced proliferation, migration, invasion and survival were mediated through PTP1B.

### 2.4. IL-13 Signaling through IL13Rα2 Requires PTP1B for Src Activation

**Figure 4:**
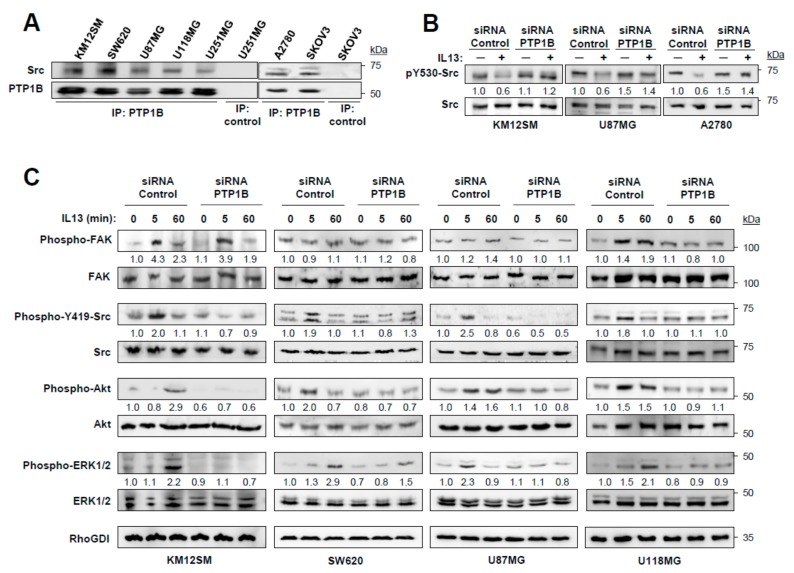
IL-13 signaling is mediated by PTP1B via Src activation. (**A**) Analysis of Src and PTP1B association by immunoprecipitation and Western blot. (**B**) KM12SM, U87MG and A2780 cells were transfected with control or PTP1B-targeted siRNAs, exposed to IL-13, lysed and the extracts analyzed by Western blot to detect pSrc Y_530_ and total Src. (**C**) KM12SM, SW620, U87MG and U118MG cells transfected with the same siRNAs were exposed to IL-13 for the indicated times and the cell extracts were analyzed for FAK, Src, AKT and ERK1/2 phosphorylation by Western blot. RhoGDI was used as loading control.

First, we confirmed the presence of Src in the PTP1B immunoprecipitates of the three types of cancer (Figure 4A). Then, we tested the phosphorylation of Src Tyr_530_ in PTP1B-silenced cells. Whereas addition of IL-13 reduced the phosphorylation of Src Tyr_530_ in control cells, the phosphorylation levels in PTP1B-silenced cells remained constant (Figure 4B). As pSrc Tyr_530_ prevents the phosphorylation of pSrc Tyr_419_, we investigated the effect of knocking down PTP1B on Src Tyr_419_ phosphorylation in the three tested cell lines and, consequently, on FAK, AKT and ERK1/2 activation at different times. pSrc Tyr_419_ activation was an early event (5 min) that was suppressed by knocking down PTP1B and the consequent phosphorylation of Src Tyr_530_ (Figure 4C). FAK phosphorylation was not affected by PTP1B silencing, except in U118 GBM cells (Figure 4C). The increase of AKT and ERK1/2 phosphorylation occurred between 5–60 min in the tested cell lines and was also decreased after PTP1B silencing (Figure 4C). In summary, PTP1B-silencing inhibited the Src/AKT/ERK pathway activation induced by IL-13 binding to IL13Rα2 in cancer cells.

### 2.5. Claramine, a PTP1B Inhibitor, Reduces Cell Migration, Invasion, Proliferation and Survival

**Figure 5:**
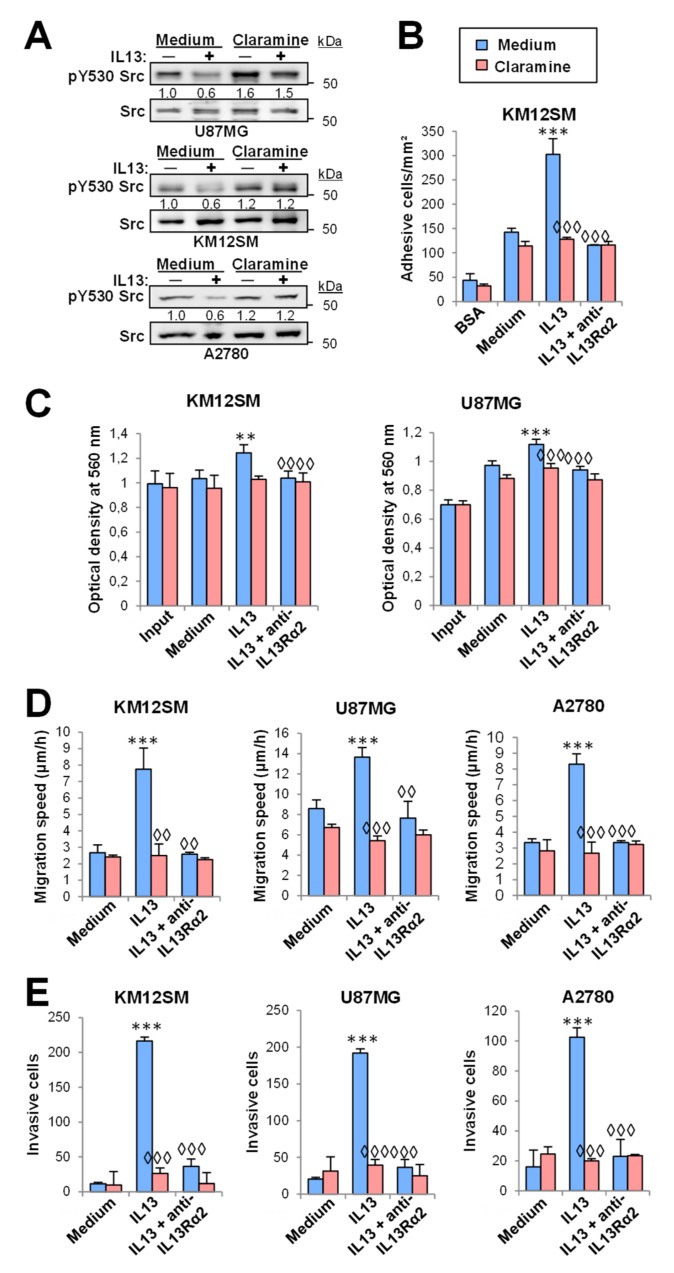
Claramine inhibits cell adhesion, migration, invasion and proliferation triggered by IL-13. (**A**) U87MG, KM12SM and A2780 cells, treated with or without Claramine, were exposed to IL-13, lysed and the extracts analyzed by Western blot to detect pSrc Y_530_ and total Src. (**B**) KM12SM cells were subjected to cell adhesion. (**C**) KM12SM and U87MG cells were subjected to MTT assays. The three cell lines were subjected to (**D**) migration and (**E**) invasion assays. All the experiments were done in the presence or absence of IL-13, Claramine and/or anti-IL13Rα2 blocking antibodies. Cell adhesion/migration/invasion and proliferation were significantly increased by addition of IL-13 (** *p* < 0.01; *** *p* < 0.001) and inhibited by treatment with Claramine or the blocking antibody (◊◊ *p* < 0.01; ◊◊◊ *p* < 0.001).

Next, we investigated the effect of the PTP1B inhibitor, Claramine, on the pro-invasive effects of IL-13. First, we studied the cellular toxicity of Claramine at different doses. Claramine at 5 μM caused around 50% decrease in cell survival, but had no effect at 2 μM, which was used in the remaining experiments (Supplementary Figure S4). Treatment with 2 μM Claramine preserved the phosphorylation of Src Tyr_530_ in IL13-treated U87MG, KM12SM, and A2780 cells (Figure 5A). The effects of Claramine on cell adhesion, migration, invasion, and, at a minor extent, proliferation mimicked the effects of PTP1B silencing in the three types of cancer cells (Figure 5B–E). In colorectal cancer, treatment with Claramine abolished IL-13-induced cell adhesion to Matrigel at a similar extent to the use of a blocking IL13Rα2 antibody (clone 47) [36], suggesting that both treatments are likely blocking the same pathway (Figure 5B). Proliferation was also inhibited by Claramine in KM12SM and U87MG (Figure 5C). The most significant effects of Claramine were observed in its capacity to inhibit cell migration and invasion in the three types of cancer, in a similar way to the IL13Rα2 antibody (Figure 5D,E). We also noticed a significant reduction in IL-13-promoted cell survival after treatment with Claramine (Supplementary Figure S5). Together, these results support the functional relevance of PTP1B inhibitors for blocking the IL13/IL13Rα2 signaling pathway.

### 2.6. Effect of Claramine on EGF, IRS-1 and Glucose Homeostasis

**Figure 6:**
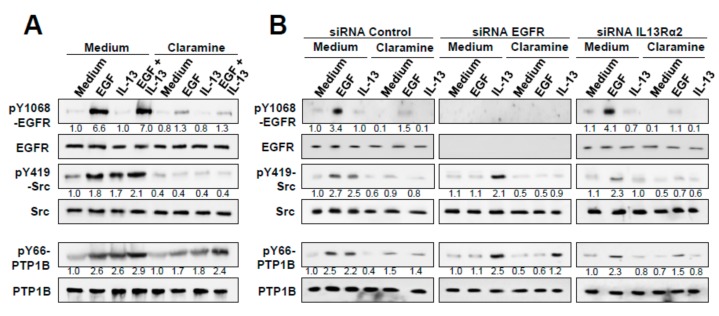
Effect of Claramine on EGF and IL-13 signaling in GBM cells. (**A**) U251 cells were treated with EGF, IL-13 or both, with or without Claramine. Cell extracts were analyzed by Western blot to detect pEGFR Y_1068_, pPTP1B Y_66_, and pSrc Y_419_. (**B**) U251 cells silenced for EGFR, IL13Rα2 or control cells were treated with EGF or IL-13 in the presence or absence of Claramine. Cells were lysed and whole extracts were analyzed by Western blot to detect pEGFR Y_1068_, pPTP1B Y_66_ and pSrc Y_419_.

EGFR is a substrate of PTP1B [37] and might cooperate with IL13Rα2 in GBM invasion [38]. To determine whether PTP1B participates in the modulation of the EGFR pathway, in addition to the IL13Rα2, we investigated the effect of Claramine on EGF and IL-13 signaling in GBM. In U251 GBM cells, phospho-EGFR Tyr_1068_ was only activated by EGF (Figure 6A), while Src Tyr_419_ phosphorylation was indistinctly triggered by EGF or IL-13. PTP1B Tyr_66_ was also phosphorylated by EGF and IL-13, indicating the participation of PTP1B in both pathways. Claramine inhibited the activation of EGFR and Src by EGF and IL-13, blocking the phosphatase active site independently of the PTP1B activation (Figure 6A). The combination of both EGF and IL-13 retained a significant PTP1B Tyr_66_ phosphorylation suggesting an additive effect. However, EGFR-silencing, did not reduce PTP1B and Src activation mediated by IL-13. Similarly, IL13Rα2-silencing did not inhibit EGFR/PTP1B/Src activation by EGF. In contrast, Claramine suppressed both, EGFR and Src activation (Figure 6B). Taken together, these data confirm that inhibition of PTP1B by Claramine might be an efficient strategy to block not only IL13 but EGF-promoted Src activation in GBM cells, increasing the potential therapeutic value of PTP1B inhibitors.

As PTP1B silencing or Claramine treatment might modulate glucose and energy homeostasis including insulin signaling, we also investigated the effect of IL-13 on the glucose uptake and insulin signaling. IL-13 increased the glucose uptake in GMB cells but not in CRC cells (Supplementary Figure S6A). However, the use of Claramine did not inhibit this uptake in GBM cells. Then, we studied the phosphorylation of insulin receptor substrate 1 (IRS-1) in CRC and GBM cells treated with IL-13 and Claramine. We found a strong phosphorylation of IRS-1 in U251 cells treated with IL-13. In contrast, phosphorylation of IRS-1 in KM12SM cells was much weaker, without differences after IL-13 addition. However, the inhibition of PTPB1 with Claramine did not affect IRS-1 phosphorylation in none of the cell lines (Supplementary Figure S6B). Together, these studies support that Claramine effect on tumor cells seems to be independent of the glucose homeostasis.

### 2.7. Claramine Increases Survival to Metastasis and Invasion in CRC and GBM Mouse Models

**Figure 7:**
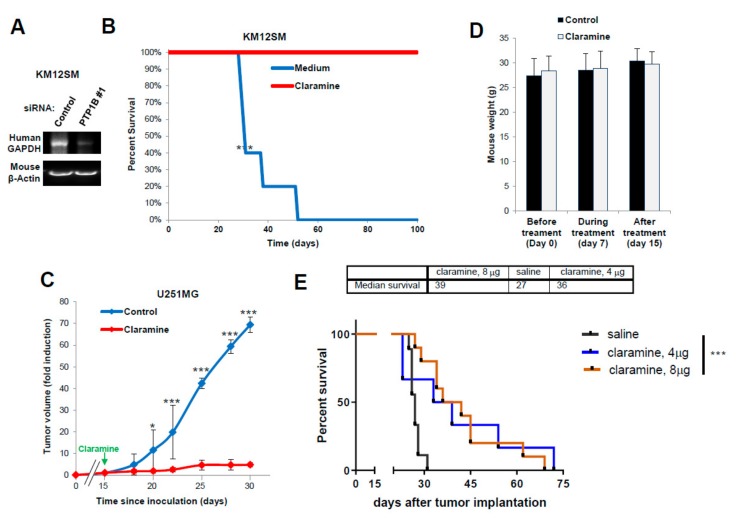
PTP1B inhibition increases survival in CRC and glioblastoma mouse models. (**A**) RT-PCR assays of RNA isolated from livers to detect human GAPDH as a surrogate of liver colonization by KM12SM cells previously transfected with the indicated siRNAs. Murine β-actin amplification was used as a loading control. (**B**) Kaplan–Meier survival analysis for mice inoculated intrasplenically with metastatic KM12SM cells and treated with Claramine. Treatment significantly increased mice survival (*** *p* < 0.001). (**C**) Mice were subcutaneously inoculated with U251 cells and treated with PBS or Claramine after 15 days of implantation. Claramine significantly inhibited tumor growth (* *p*< 0.05; *** *p* < 0.001). (**D**) Mouse weight during the treatment with Claramine remained constant. (**E**) Kaplan–Meier survival analysis for mice inoculated intracranially with GBM12 PDX cells. Treatment with Claramine started 7 days after tumor implantation and significantly increased mice survival (*** *p* < 0.001).

Finally, we investigated the in vivo effects of PTP1B silencing or inhibition in colorectal cancer and glioblastoma mouse models. Given the high homology between murine and human IL-13, we hypothesized that it was unnecessary to treat the mice with exogenous human IL-13 [11]. For a further confirmation, we compared human and murine IL-13 in an invasion experiment. Both IL-13 promoted a similar pro-invasive capacity on human cancer cells (Supplementary Figure S7). Then, we examined the effect of PTP1B silencing on the capacity for liver homing of KM12SM cells inoculated in the spleen of nude mice. As a surrogate marker for homing, human GAPDH was detected in the livers of mice inoculated with control cells but not in those inoculated with PTP1B-silenced cells (Figure 7A). Then, we investigated the effects of Claramine on mice survival in colorectal cancer metastasis. Forty-eight h after spleen inoculation with CRC cells, mice were treated intraperitoneally (i.p.) with Claramine for two weeks on alternate days, with a total dose of 49 µg/mouse. Treated mice were sacrificed at day 100 post-inoculation without symptoms of the disease, the presence of metastatic nodes or weight loss. Kaplan–Meier survival curves showed that Claramine caused complete protection against liver metastases in the treated mice (Figure 7B).

Next, we investigated the in vivo effect of Claramine on GBM tumor growth and intracranial inoculation. First, mice were subcutaneously inoculated with U251 cells and xenografts were allowed to grow for 15 days before starting i.p. treatment with Claramine (Figure 7C). Tumors in treated mice stop growing for the duration of the treatment when compared with non-treated mice. No Claramine toxicity was observed in the treated animal, as indicated by the constant weight of the animals (Figure 7D). In addition, we studied the involvement of IL13Rα2 and PTP1B in the tumor growth using xenograft tissues. We found a significant increase of PTP1B phosphorylation (3–5 times) in vivo compared with cells in culture (Supplementary Figure S8). Treatment with Claramine provoked a moderate inhibition in the phosphorylation of PTP1B, as previously observed in cultured cells (Figure 6A). We also noticed an increased expression of IL13Rα2 in the xenografts (Supplementary Figure S8). These results indicate a correlation between increased IL13Rα2 expression and PTP1B activation associated to tumor growth.

Finally, we tested an intracranial model of GBM using the GBM12 PDX that expresses high amounts of IL13Rα2. Tumors were implanted intracranially for seven days before starting the treatment with four doses of Claramine for a total of 4 mg/kg or 8mg/kg (Figure 7E). The median survival for the treated mice increased up to 36–39 days from 27 days of the control mice. Treated mice survived up to 70–72 days post-inoculation and Kaplan–Meier analysis showed a statistically significant difference in survival between treated and control groups (*p* < 0.001). In summary, these data demonstrate the efficacy of Claramine for improving glioblastoma and colorectal cancer survival in mouse models.

## 3. Discussion

In this report, we discovered the association between IL13Rα2 and the tyrosine phosphatase PTP1B for IL-13 signaling in different cancer types, paving the way to the therapeutic use of PTP1B inhibitors. Our main findings were that (i) PTP1B was present in the IL13Rα2 interactome network, (ii) PTP1B was shown to interact with the cytoplasmic tail of IL13Rα2 through phospho-Tyr_369_, (iii) IL-13 triggered PTP1B-mediated activation of Src followed by activation of PI3K/AKT and ERK pathways, (iv) Claramine, a PTP1B inhibitor, abolished the IL-13 pro-invasive and pro-metastatic effects and (v) Claramine caused a complete inhibition of CRC liver metastasis in Swiss nude mice, the regression of GBM xenografts and a considerable increase of mice survival after intracranial inoculation of PDX GBM cells. In summary, we provide strong evidence that PTP1B inhibition is a promising strategy for cancers overexpressing IL13Rα2, such as advanced colorectal, ovarian, and glioblastoma, among others.

PTP1B-mediated Src activation took place either after IL-13 or EGF binding to their receptors in an independent way. GBM cells may use either EGF and/or IL-13 to promote invasion as both pathways rely on PTP1B for Src activation. Other proteins present in the IL13Rα2 network were CSNK1A1 (casein kinase I), LZIC, PBK (PDZ binding kinase) and PDZD8 (PDZ Domain-Containing Protein 8). CSNK1A1 has been involved in the modification of substrates for different signaling pathways [39]. PBK promotes migration in lung cancer by modulating the PI3K/AKT pathway [40]. PDZD8 is known to tether endoplasmic reticulum (ER) and mitochondria in mammalian cells [41], and might be linked to the reported PTP1B-ER contacts.

PTP1B is targeted to the endoplasmic reticulum (ER) membrane via a hydrophobic sequence at its C-terminus [42]. The mechanism by which ER-bound PTP1B interacts with plasma membrane proteins, like IL13Rα2, is not completely understood [22]. It has been proposed that PTP1B recognizes receptor tyrosine kinases when they are internalized after ligand binding and endosomes come in close contact with the endoplasmic reticulum (ER). As IL13Rα2 follows a similar internalization pathway [20], it might be recognized in a similar way. Alternatively, PTP1B might be present in a dynamic ER membrane that could contact the substrates at the plasma membrane [43]. Other mechanisms are also plausible, including protein interaction after “de novo” biosynthesis. As PTP1B has been involved in glucose homeostasis and insulin signaling, we tested the role of IL13 on glucose uptake and insulin signaling. Claramine did not affect glucose uptake by the GBM cells and had no effect on IRS-1 phosphorylation, suggesting that the anti-tumoral effect of PTP1B inhibition in these cancer cells relies mainly in Src inhibition and not on glucose homeostasis. As a key regulator of the insulin and leptin signaling pathways, PTP1B has been thoroughly investigated as a target for the development of clinically useful inhibitors. Claramine is an insulin-mimetic compound that displays a selective inhibition for PTP1B but not for the closely related phosphatase TC-PTP. Here, Claramine was extremely effective in blocking the liver colonization and metastatic growth in CRC as none of the treated mice developed metastatic lesions. In addition, Claramine inhibited the growth of GBM xenografts during the course of treatment. The final proof of the Claramine efficacy in GBM was shown in an intracranial GBM model using a highly aggressive IL13Rα2-expressing GBM12 PDX line. The results are very encouraging as we observed an almost 50% increase in half-life of the mice. Claramine treatment was initiated 7 days after inoculation of the tumor cells and were concentrated in only four small doses. As a reference, after irradiation, a standard of care for GBM patients, there was no survival improvement in animals inoculated with the same GBM12 intracranial xenograft [44].

The development of improved drug carriers and advanced delivery system would be desirable. Our results suggest that the treatment might require a continuous delivery of the drug to tumor for eradication or for preventing the tumor’s progression. Moreover, we have to take into consideration that the administration of free drug is likely not the optimal system for local delivery. Examples of systems tailored to improve the efficacy of the Claramine for a more efficient administration and cellular entry should include liposomes, nanoparticles or micropumps. Although the capacity of Claramine to suppress feeding behavior in mice suggested that Claramine might cross the blood-brain barrier [45], our data in the GBM intracranial models have demonstrated that Claramine likely does not cross the blood–brain barrier in the effective dose (Supplementary Figure S9).

Still, multiple questions, including the capacity of Claramine to inhibit IL13Rα2 negative tumors or the potential synergy with chemotherapeutic drugs like temozolomide or to overcome temozolomide-resistant tumors in GBM remain unanswered and will require further experimentation. At the indicated doses, Claramine did not show any sign of toxicity in treated mice, including changes in the behavior of animals or in the body weight (Figure 7D). In any case, a more exhaustive characterization of the Claramine safety issues will be necessary in future work. However, Claramine was used as a proof-of-concept of the capacity of PTP1B inhibitors to arrest tumor growth and metastasis in multiple cancers. Other PTP1B inhibitors currently in development [46,47] could be equally or more effective than Claramine.

## 4. Materials and Methods

### 4.1. Cell Culture, PDXs, and Reagents

Highly metastatic KM12SM human colon cancer cells were obtained from Dr. I. Fidler (MD Anderson Cancer Center. Houston, TX, USA). U87MG and U118MG glioblastoma cell lines were provided by Dr. G. Velasco (UCM, Madrid, Spain). Ovarian cancer cells A2780 were provided by Dr. F. Diaz-Pereira (CIB) and SKOV3 by Dr. A.I. Torres (UCM). Human SW480, SW620 and RKO colon cancer, OVCAR3 ovarian and U251MG GBM cell lines were purchased from the ATCC and passaged less than 6 months after purchase for all the experiments. All cell lines were cultured in DMEM (Invitrogen) containing 10% FCS (Invitrogen) and antibiotics at 37 °C in a 5% CO_2_ humidified atmosphere. Tumor sample lysates from GBM6, GBM43 and GBM12 PDX glioblastoma lines were kindly provided by Dr. C.D. James (Northwestern University, Chicago, USA).

Human and murine IL-13 were used at 10 ng/mL and EGF at 50 ng/mL. These proteins were purchased from PeproTech. Claramine (Sigma-Aldrich, St. Louis, MO, USA) was used at 2 µM for in vitro assays and 49–200 µg/mice for in vivo assays. Antibodies used in the experiments listed in Supplementary Table S2.

### 4.2. Mutagenesis of IL13Rα2 and siRNA Transfections

For IL13Rα2 Tyr_369_(TAC)-Phe(TTC) mutagenesis, vectors encoding for full-length IL13Rα2 (IMAGE collection) were subjected to mutagenesis using QuickChange Lightning Site-Directed Mutagenesis Kit (Agilent Technologies, Santa Clara, CA, USA) using oligonucleotides: 5′-CTTTTGCGTAAGCCAAAGA-GGTTCCCAAAAATGATTCCA-3′ and 5′-AAAATTCTGGAATCATTTTT-GGGAAGGTGTTTGGCTTACGC-3′ according to the manufacturer’s instructions. Mutation was confirmed by in-house sequencing. Different siRNAs targeting PTP1B (#1 SASI-Hs01-00230698 and #2 SASI-Hs02-00334527), EGFR (5′-UGUGCCACCUGUGCCAUCCdTdT-3′) or IL13Rα2 [10] were obtained from Sigma-Aldrich. siRNAs, as well as the vectors encoding for wild type or mutated IL13Rα2 were transfected with JetPrime (Polyplus Transfection, New York, NY, USA).

### 4.3. Western Blot

Western blot assays were done as previously described [20].

### 4.4. Immunoprecipitation and Mass Spectrometry

Immunoprecipitation (IP) assays were done as previously described [20]. For mass spectrometry (MS) analysis, 2 mg of cell lysates were subjected to immunoprecipitation and loaded in SDS–PAGE. Mass spectrometry conditions and analyses were carried out as previously described [20].

### 4.5. Adhesion, Wound Healing and Invasion Assays

Adhesion, wound healing and invasion assays have been previously reported [21].

### 4.6. MTT Assays for Proliferation Assessment and for Survival to Oxidative Stress

Proliferation assays have been previously described [21]. To assess cell survival to oxidative stress, 10^4^ cells were seeded per well on 96-well plates and incubated for 24 h in DMEM containing 1% serum in presence of 1mM H_2_O_2_, followed by 1 h incubation with MTT. Cell viability was determined by A_560nm_ and compared with untreated cells.

### 4.7. Flow Cytometry, Internalization and Glucose Uptake Assays

Flow cytometry was performed as previously described [20]. For IL13Rα2 internalization assays, cells were starved for 3 h, detached with 2 mM EDTA, incubated 30 min with IL-13 at 37 °C, primary and secondary antibodies for 30 min at 4 °C and analyzed in the cytofluorimeter as previously described [21]. For glucose uptake determination, cells were incubated with IL-13 and/or Claramine and subjected to the assays using 2-NBDG glucose uptake assays kit (BioVision, Milpitas, CA, USA) according to the manufacturer’s instructions. Glucose uptake was quantified in the cytofluorimeter.

### 4.8. In Vivo Animal Experiments

The Ethics Committee of the Consejo Superior de Investigaciones Científicas (Madrid, Spain) and the Community of Madrid approved the protocols used for xenograft and intra-spleen injections with mice. For GBM xenografts, NSG mice were subcutaneously inoculated with U251 cells. Then, mice were treated intraperitoneally with 7 doses of Claramine (every three days) starting day 15 after implantation (a total of 2 mg/kg of body weight). The size of subcutaneous tumors was measured every 2 days. After euthanasia, tumors were isolated and the extracts subjected to Western blot analysis to determine IL13Rα2 and PTP1B activation. Liver homing and metastasis experiments in Swiss nude mice have been described [20]. For CRC metastasis, mice were treated with the same dose of Claramine as above. Animals were weighed to study the effects on food intake and tolerance to the treatment.

For the intracranial experiments, athymic nude male mice were purchased from Jackson Laboratories. All the experiments were approved by the Northwestern University Institutional Animal Care and Use Committee (IACUC). For the intracranial implantation of cannula and tumor cells, 6–8 weeks old female mice were first anesthetized with a ketamine HCl (25 mg/mL)/xylazine (2.5 mg/mL) cocktail. A previously established surgical procedure was utilized in this study with a small variation reflecting the cannula implantation [48]. For that, a custom-made 26-gauge sterile guide cannula (Plastics One, Roanoke, VA, USA) was installed into the brain through a burr hole at a 2 mm depth and secured with tissue glue (3M, St Paul, MN, USA). For the glioma PDX cells implantation, a 33-gauge sterile syringe was inserted into the guide cannula at 3 mm depth following by infusion of 2.5 µL of 10^5^ GBM12 cells. The skin incision was closed with surgical glue around the implantation site. The extracranial end of the cannula was then covered with a 33-gauge protection dummy cannula. The surgical procedure was followed with a standard post-surgery care according to the approved protocol. The intracranial injection of Claramine through cannula was done in 4 fractions for a total 4–8 mg/kg starting day 7 after tumor implantation and every 3 days after that. For systemic treatment, Claramine was delivered intravenously in 4 fractions for a total 8, 15 or 30 mg/kg starting day 7 after tumor implantation and every 3 days after that. Sterile 0.9% saline solution served as a negative control in all experiments.

### 4.9. In Silico Expression and Prognostic Studies

The GSE17538 database, which contains 244 tumor samples with clinicopathological data, was used for the prognostic study in CRC. Data were normalized using Bioconductor’s Affymetrix package. The prognostic value of PTP1B expression level was assessed using Kaplan–Meier survival curves, where negative and positive z-scores were considered as low and high expression, respectively. For glioma tumors, PTP1B association with prognosis was evaluated using the REMBRANDT dataset (containing 329 samples) using the median as a threshold to divide into low and high expression populations. For ovarian cancer, PTP1B association with survival was assessed using the GEPIA2 dataset (gepia.cancer-pku.cn, accessed on 15 July 2019 containing 468 ovarian cancer samples. They were analyzed by considering as threshold for high PTP1B expression the 40% most positive samples.

### 4.10. Statistical Analyses

At least three replicates were done for each experiment. Data were analyzed by one-way ANOVA followed by Tukey–Kramer multiple comparison test. The significance of the difference for survival curves was estimated with the log-rank test. In all analyses, the minimum acceptable level of significance was *p* < 0.05.

## 5. Conclusions

We uncovered the relevance of PTP1B-mediated Src activation for IL-13 signaling through IL13Rα2 in multiple types of cancer. IL-13/IL13Rα2 activation of PTP1B was independent of EGFR activation and was critical for migration and invasion of cancer cells. Moreover, our results provide us with a complete picture of the IL-13/IL13Rα2 signaling pathway upstream of the transcriptional regulation induced by AP-1. Remarkably, treatment with Claramine, a PTP1B inhibitor, completely blocked metastatic colonization in CRC, and resulted in a significant survival advantage in an aggressive intracranial model of GBM. Based on these results, we propose PTP1B inhibition as a therapeutic target for the treatment of metastatic colorectal and ovarian cancer as well as for glioblastoma therapy. The plausible extension to other IL13Rα2-expressing cancers increases the value of PTP1B inhibitors as potential therapeutic agents for cancer invasion and metastasis.

## Supplementary Materials

The following are available online at https://www.mdpi.com/2072-6694/12/2/500/s1, Figure S1: PTP1B regulates IL13Rα2 expression levels in cell membrane. Figure S2. Silencing of PTP1B inhibits IL-13-triggered cell adhesion, migration, invasion and proliferation in cancer cells. Figure S3. PTP1B is required for IL-13-induced promotion of cell survival to oxidative stress. Figure S4. Claramine affects cell survival in a dose-dependent fashion. Figure S5. PTP1B is required for IL-13-induced promotion of cell survival to oxidative stress. Figura S6. Effect of Claramine on glucose homeostasis and IRS-1 activation. Figure S7. Murine IL-13 is functionally equivalent to human IL-13 in IL13Rα2 expressing human cancer cells. Figure S8. Expression of PTP1B and IL13Rα2 in tumor xenografts recovered after subcutaneous inoculation of U251MG in mouse. Figure S9. Claramine does not cross the blood-brain-barrier. Table S1: IL13Rα2 co-immunoprecipitated proteins in U251 glioblastoma cells. Table S2: Antibodies used in the different applications.
