# Supplementary material for: Protein Tyrosine Phosphatase-1B Inhibition Disrupts IL13Rα2-Promoted Invasion and Metastasis in Cancer Cells
Source: Cancers (Basel). 2020 Feb 21;12(2):500. doi: 10.3390/cancers12020500 (PMC7072372; doi:10.3390/cancers12020500)
Supplement: Supplementary file 1 [file cancers-12-00500-s001.zip › suppl-final/cancers-726732-suppl-final.docx]

Article

Protein Tyrosine Phosphatase-1B Inhibition Disrupts IL13Rα2-Promoted Invasion and Metastasis in Cancer Cells

Rubén A. Bartolomé, Ángela Martín-Regalado, Marta Jaén, Markella Zannikou, Peng Zhang, Vivian de los Ríos, Irina V. Balyasnikova and J. Ignacio Casal

Supplementary Materials

**Figure S1.** PTP1B regulates IL13Rα2 expression levels in cell membrane. (A) Flow cytometry analysis showed the expression of IL13α2 in the surface of KM12SM and U87 cancer cells transfected with control or PTP1B-targeted siRNAs and treated with IL-13. Mean fluorescence intensity is shown inside each panel. (B) Western blot of IL13α2 in the same transfectants of A. RhoGDI was used as loading control (C) KM12SM and U87 cells were transfected with siRNAs control or targeting IL13Rα2. Silenced cells were treated with IL-13 for 24 h, lysed and the extracts analyzed by Western blot to assess the expression of PTP1B (left). Knock down of IL13Rα2 was confirmed by Western blot analysis of both transfectants (right).

**Figure S2.** Silencing of PTP1B inhibits IL-13-triggered cell adhesion, migration, invasion and proliferation in cancer cells. (A) Cancer cells were transfected with control siRNA or with different PTP1B-targeting siRNAs. Verification of silencing was done 48 h after transfection by Western blot. The same transfectants were subjected to cell adhesion assays (B), MTT assays (C), migration assays (D) or invasion assays (E) in presence of IL-13. Cell adhesion/optical density/migration/invasion was significantly increased by addition of IL-13 (*, *p* < 0.05; **, *p* < 0.01; ***, *p* < 0.001) and decreased by PTP1B silencing (◊, *p* < 0.05; ◊◊, *p* < 0.01; ◊◊◊, *p* <0.001).

**Figure S3.** PTP1B is required for IL-13-induced promotion of cell survival to oxidative stress. Cancer cells were transfected with the indicated siRNAs and subjected to survival assays using 1mM H2O2 for 24 h in the presence or absence of IL-13. The presence of IL-13 induced an increment of viable cells (**, *p* < 0.01; ***, *p* < 0.001), whereas the transfection of siRNAs against PTP1B inhibited the promotion of cell survival by IL-13 (◊◊, *p* < 0.01; ◊◊◊, *p* < 0.001).

**Figure S4.** Claramine affects cell survival in a dose-dependent fashion. MTT assays were carried out with KM12SM and U87 cells in the presence of Claramine at the indicated concentrations for 24 (*left*) or 48 h (*right*).

**Figure S5.** PTP1B is required for IL-13-induced promotion of cell survival to oxidative stress. Cancer cells were transfected with the indicated siRNAs and subjected to survival assays using 1mM H2O2 for 24 h in the presence or absence of IL-13 and Claramine. Whereas IL-13 increased survival (** *p* < 0.01; *** *p* < 0.001), Claramine inhibited the cell survival (◊◊, p < 0.01; ◊◊◊, p < 0.001).

**Figure S6**. Effect of Claramine on glucose homeostasis and IRS-1 activation. (**A**) KM12SM and U251MG cells treated with IL-13 and/or Claramine were subjected to glucose uptake assays carried out with “2-NBDG glucose uptake assay” kit (BioVision) and quantified by flow cytometry. Addition of IL-13 significantly increased the glucose uptake (***, *p* < 0.001) in U251MG GBM cells, but did not cause significant glucose alterations in KM12SM CRC cells. Treatment with Claramine did not modify the glucose uptake in any of the cell lines. Moreover, both effects seem to be IL13Rα2-independent. (**B**) Western blot analysis of IRS-1 phosphorylation in KM12SM and U251MG cells treated with IL-13 and/or Claramine. This result suggests a role for the IL13Rα1 receptor in the activation of IRS-1. GBM cells express this receptor while KM12SM cells do not express it. Moreover, IRS-1 exhibited constitutive activation in KM12SM cells without IL-13 addition.

**Figure S7.** Murine IL-13 is functionally equivalent to human IL-13 in IL13Rα2 expressing human cancer cells. KM12SM and U251MG cells were subjected to invasion across Matrigel towards human or murine IL-13. Assays were performed in the presence or absence of 2 μM Claramine. Both IL-13, human and murine, promoted cell invasion (**, *p* < 0.01; ***, *p* < 0.001), which was inhibited by Claramine (◊◊, *p* < 0.01; ◊◊◊, *p* < 0.001).

**Figure S8.** Expression of PTP1B and IL13Rα2 in tumor xenografts recovered after subcutaneous inoculation of U251MG cells in mouse. After 15 days, mice were treated with Claramine for another 15 days. Xenografts were isolated (see Figure 7C), lysates were resolved by SDS-PAGE and analyzed by Western blot with the indicated antibodies. Cultured U251MG cells were used as reference. A significant increase in IL13Rα2 expression and PTP1B phosphorylation was observed in the xenografts respect to the cells in culture. A small decrease in pPTP1B was observed in Claramine-treated xenografts. This inhibition was similar to that observed in cultured cells (Figure 6A).

**Figure S9.** Claramine does not cross the blood-brain-barrier. Kaplan-Meier survival analysis for mice inoculated intracranially with GBM12 PDX cells and treated systemically with two different doses of Claramine 7 days after tumor implantation. No effect of Claramine on mice survival was observed.

**Supplementary Table S1.** IL13Rα2 co-immunoprecipitated proteins in U251 glioblastoma cells.

| **Function** | **Accesion** | **Name** | **Description** | **Score** | **Coverage** | **Peptides** | **PSM** |
| --- | --- | --- | --- | --- | --- | --- | --- |
| cell signaling | P18031 | PTPN1 | Tyrosine-protein phosphatase non-receptor type 1 | 69.56 | 47.13 | 15 | 19 |
| cell signaling | P48729 | CSNK1A1 | Casein kinase II subunit alpha 2 | 19.89 | 14.84 | 4 | 5 |
| cell signaling | Q5VW36 | FOCAD | Focadhesin | 12.97 | 1.83 | 3 | 4 |
| cell signaling | Q96KB5 | PBK | Lymphokine-activated killer T-cell-originated protein kinase | 13.08 | 10.56 | 3 | 4 |
| cell signaling | Q8WZA0 | LZIC | Protein LZIC | 15.95 | 11.58 | 1 | 3 |
| cell signaling | Q01970 | PLCB3 | Isoform 2 of 1-phosphatidylinositol 4,5-biphosphate phosphodiesterase beta-3 | 10.56 | 1.71 | 2 | 3 |
| cell signaling | P62993 | GRB2 | Growth factor receptor-bound protein 2 | 10.48 | 23.04 | 3 | 3 |
| cell signaling | P60983 | GMFB | Glia maturation factor beta | 10.16 | 30.28 | 3 | 3 |
| cell signaling | Q16513 | PKN2 | Isoform 3 of serien/threonine-protein kinase N2 | 9.93 | 4.27 | 2 | 2 |
| cytoskeleton | Q15511 | ARPC5 | Actin-related protein 2/3 complex subunit 5 | 13.43 | 19.14 | 3 | 4 |
| cytoskeleton | Q8NEN9 | PDZD8 | PDZ domain-containing protein 8 | 13.50 | 4.07 | 3 | 3 |

**Supplementary Table S2.** List of antibodies used in the different applications.

| **Antibody** | **Target** | **Application** | **Origin** |
| --- | --- | --- | --- |
| ab55275 | IL13Rα2 | Flow cytometry | Abcam |
| clone 47 | IL13Rα2 | Blocking | Descibed in Balyasnikova et al. 2012 J. Biol. Chem. 287:30215-30227. |
| 2K8 | IL13Rα2 | Immunoprecipitation, western blotting | Santa Cruz Biotechnologies |
| B-9 | PTP1B | Immunoprecipitation, western blotting | Santa Cruz Biotechnologies |
| PA5-64706 | phospho-PTP1B Tyr66 | Western blotting | ThermoFisher |
| A-20 | RhoGDIα | Western blotting | Santa Cruz Biotechnologies |
| D7A5 | Phospho-Tyr1068 EGFR | Western blotting | Cell Signaling Technology |
| #4695 | p44/42 MAP Kinase | Western blotting | Cell Signaling Technology |
| #9106 | phospho-Thr202-p44/ phospho-Tyr204-p42 MAPK | Western blotting | Cell Signaling Technology |
| A-17 | FAK | Western blotting | Santa Cruz Biotechnologies |
| FAK (pY397) 14 | phospho-Tyr397-FAK | Western blotting | BD Transduction Laboratories |
| AF3389 | SRC | Western blotting | R&D Systems |
| #6943 | phospho-Tyr416 Src family | Western blotting | Cell Signaling Technology |
| #2105 | phospho-Tyr530 Src | Western blotting | Cell Signaling Technology |
| #2920 | AKT | Western blotting | Cell Signaling Technology |
| #3787 | phospho-Ser473-AKT | Western blotting | Cell Signaling Technology |
| 1005 | EGFR | Western blotting | Santa Cruz Biotechnologies |
| #3777 | phospho-Tyr1066 EGFR | Western blotting | Cell Signaling Technology |
| E-12 | IRS-1 | Western blotting | Santa Cruz Biotechnologies |
| #2381 | phospho-Ser307 IRS-1 | Western blotting | Cell Signaling Technology |

| 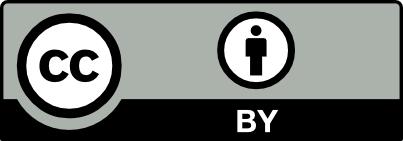 | © 2020 by the authors. Licensee MDPI, Basel, Switzerland. This article is an open access article distributed under the terms and conditions of the Creative Commons Attribution (CC BY) license (http://creativecommons.org/licenses/by/4.0/). |
| --- | --- |
