# Supplementary material for: Protein Tyrosine Phosphatase-1B Inhibition Disrupts IL13Rα2-Promoted Invasion and Metastasis in Cancer Cells
Source: Cancers (Basel). 2020 Feb 21;12(2):500. doi: 10.3390/cancers12020500 (PMC7072372; doi:10.3390/cancers12020500)

Figure 1B (right)

WB: PTP1B

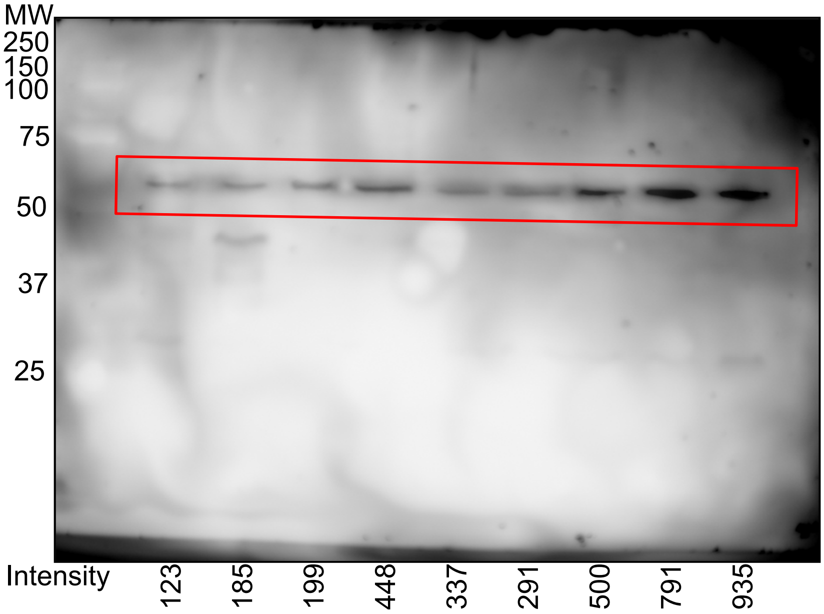

Figure 1B (left)

WB: PTP1B

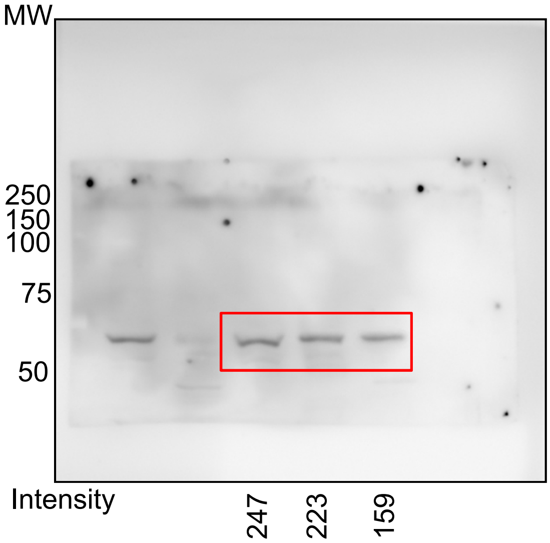

WB: RhoGDI

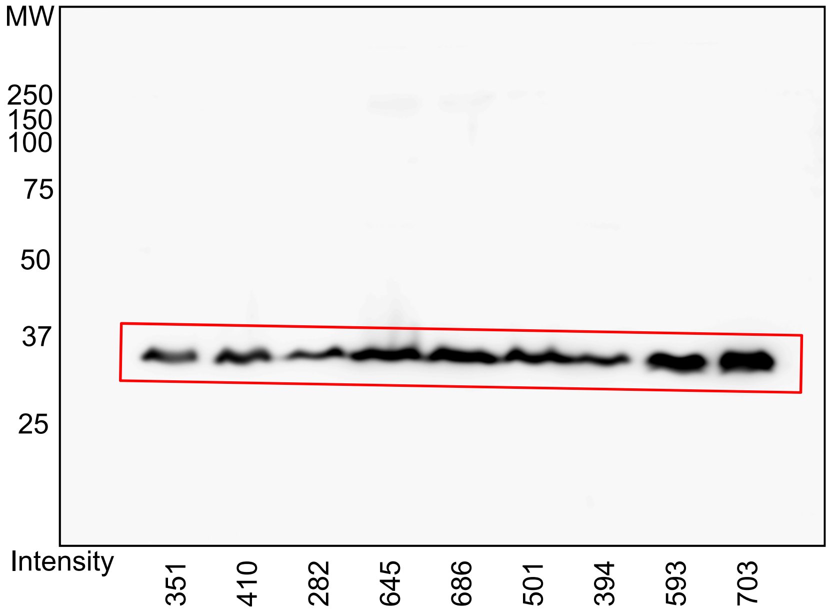

WB: RhoGDI

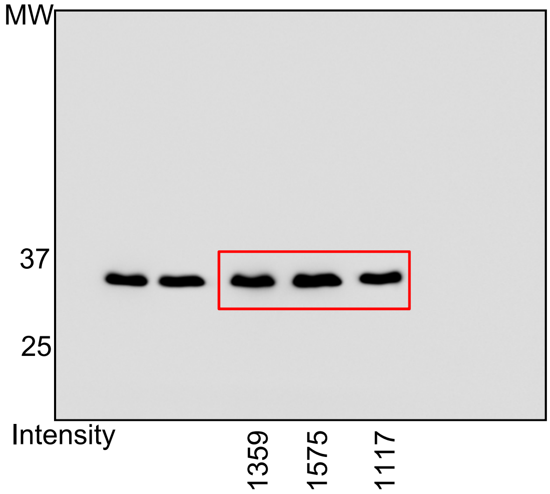

Figure 1C (up, right)

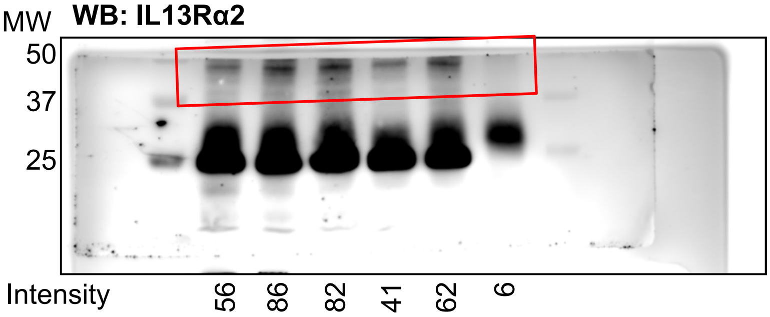

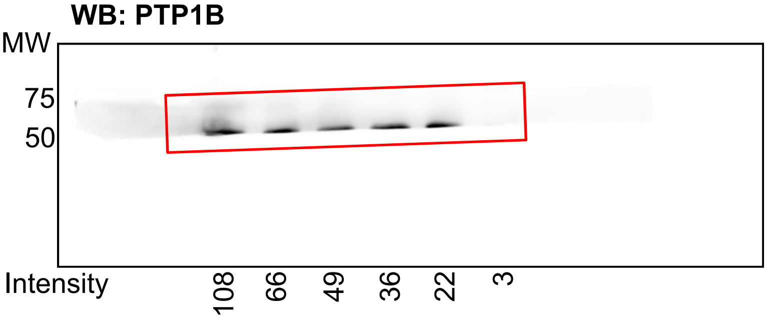

Figure 1C (up, left)

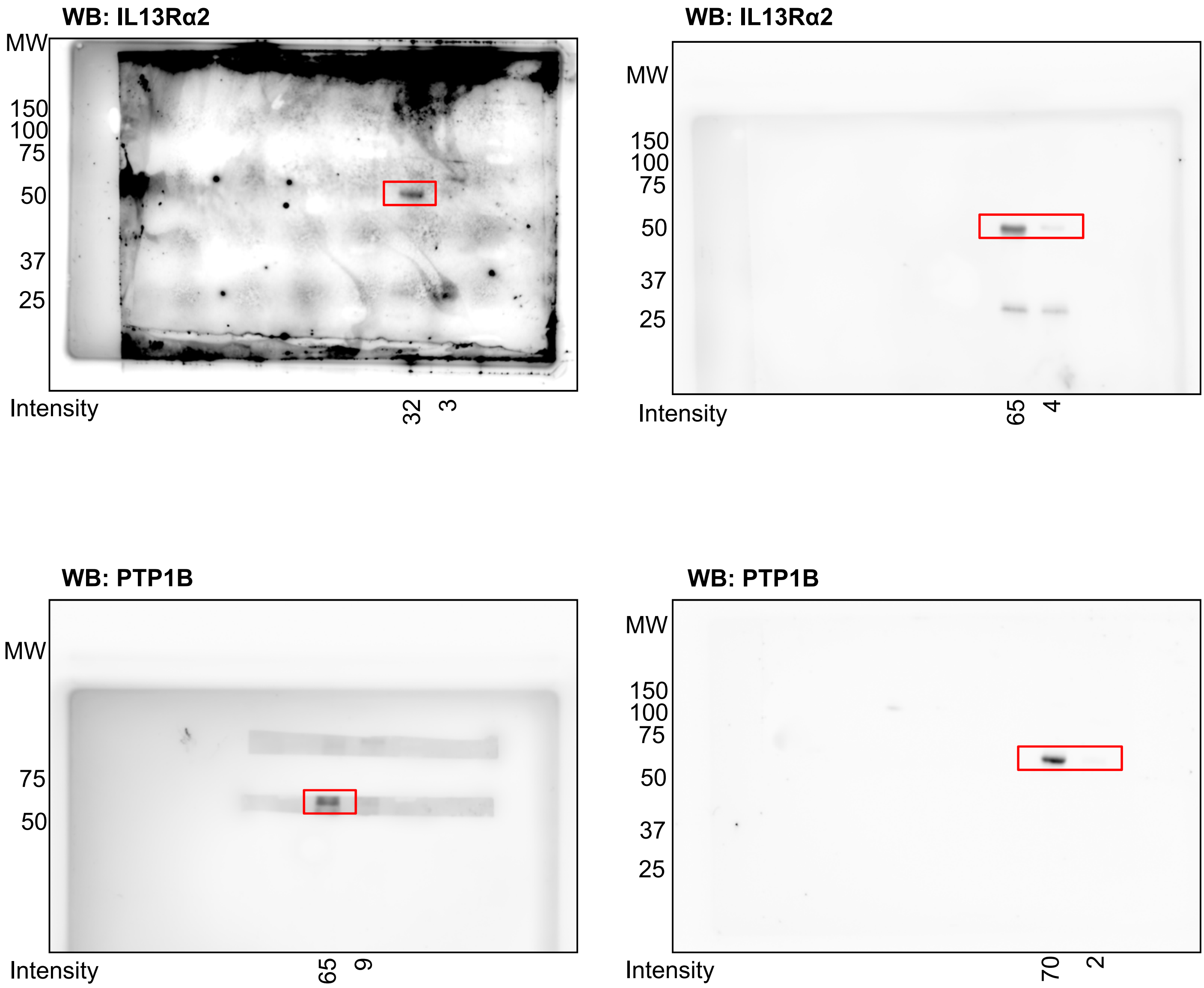

Figure 1C (down, right)

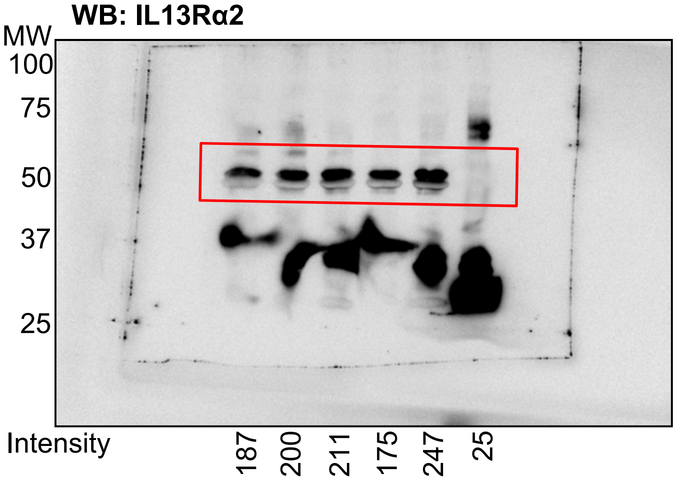

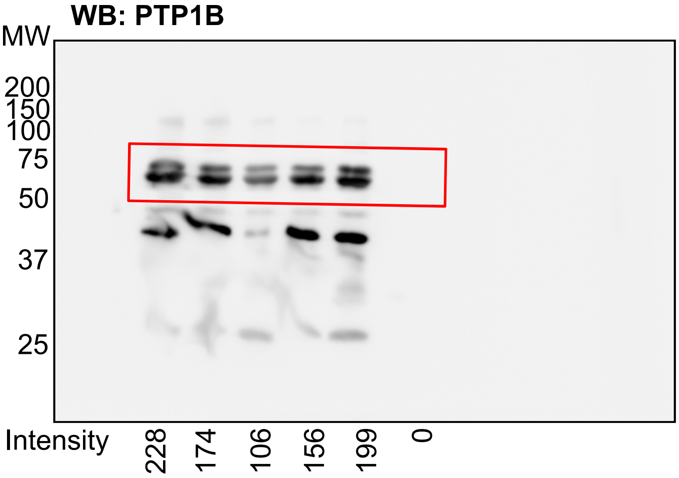

Figure 1C (down, left)

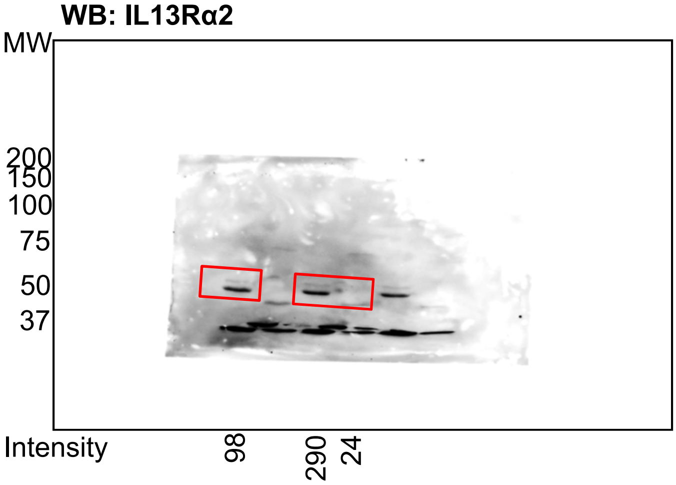

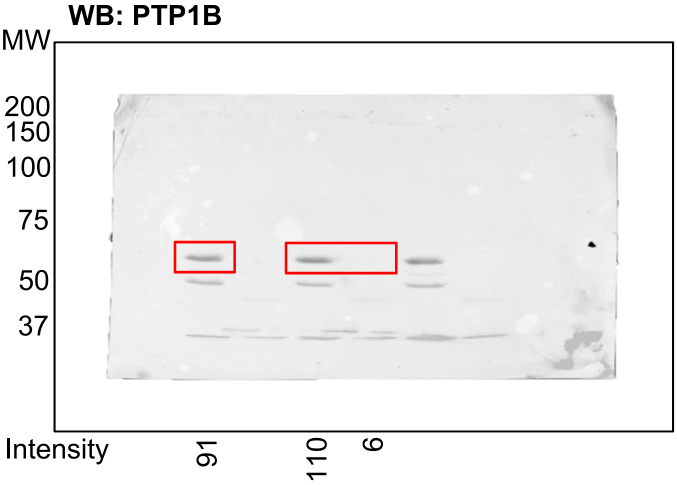

Figure 1E (up)

WB: PTP1B

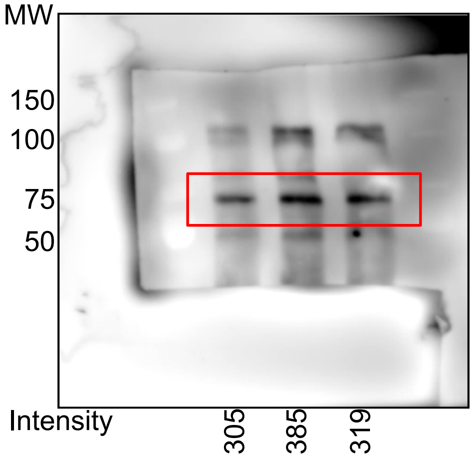

WB: IL13Rα2

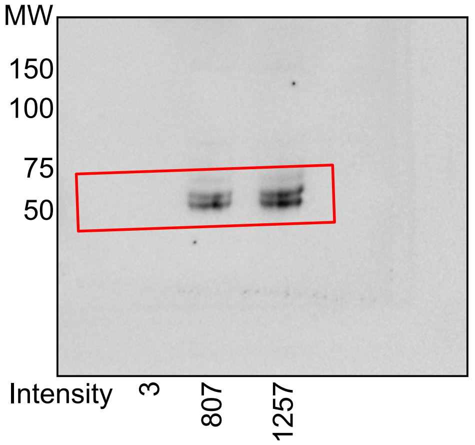

WB: RhoGDI

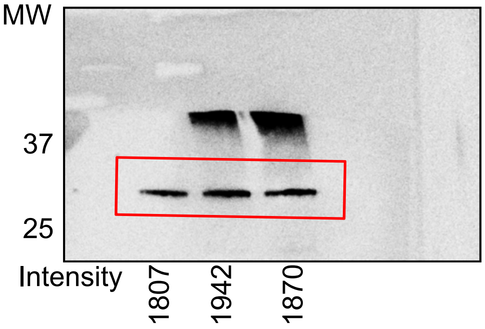

Figure 1E (down)

WB: PTP1B

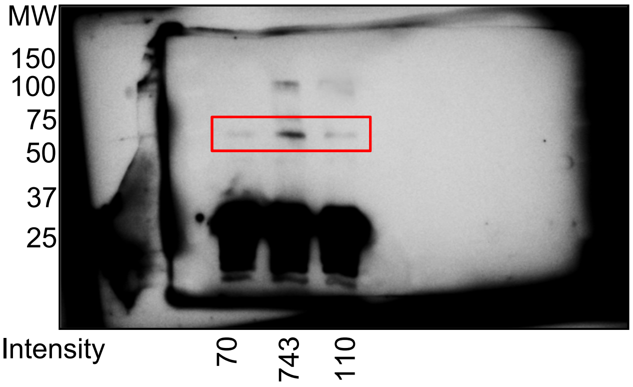

WB: IL13Rα2

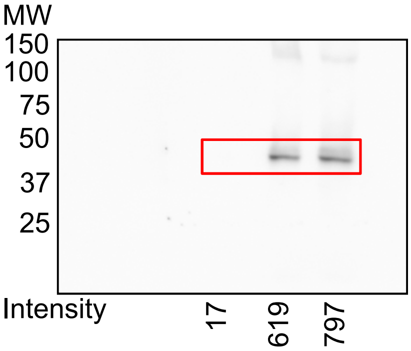

Figure 4A (left)

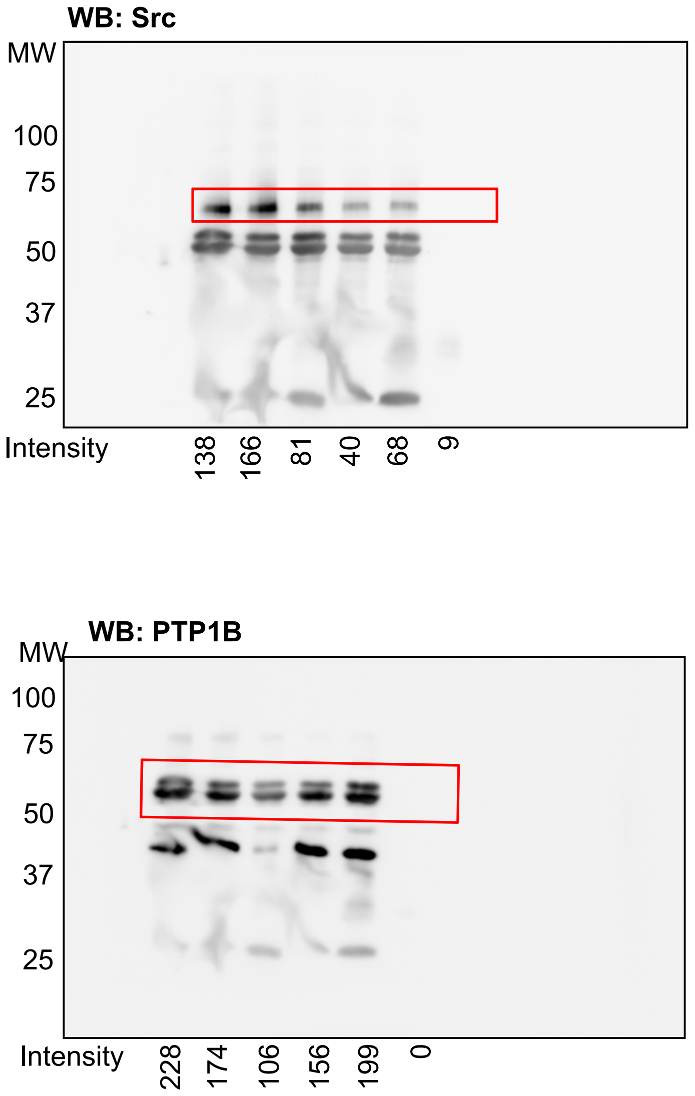

Figure 4B (center)

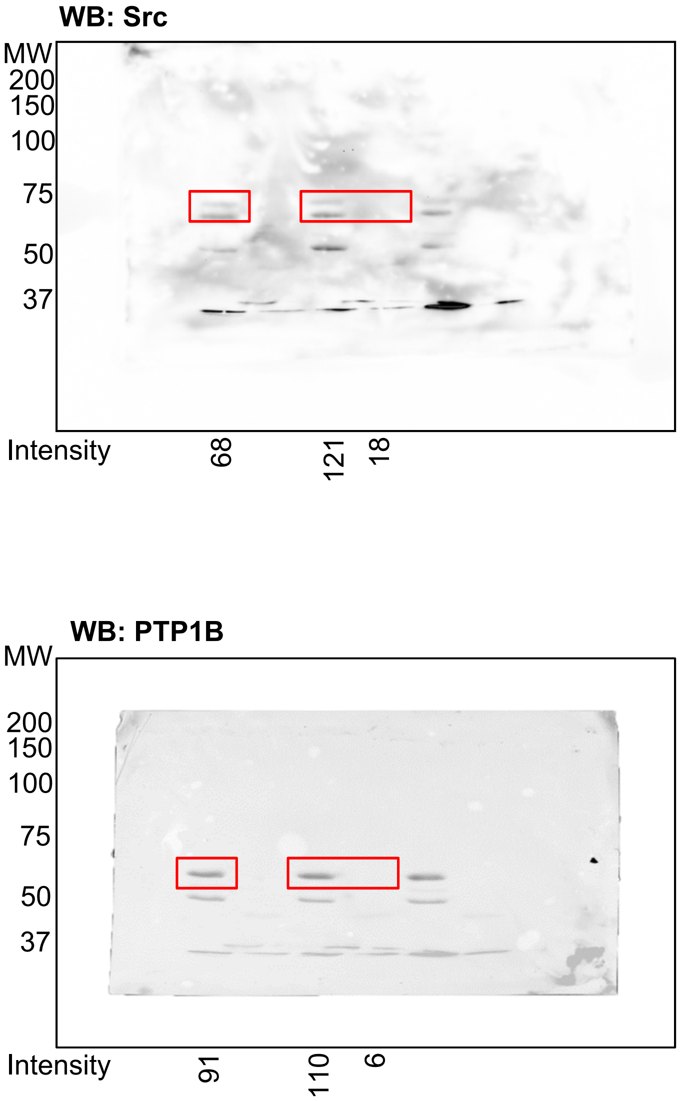

Figure 3A (up) Figure 3A (center) Figure 3A (down)

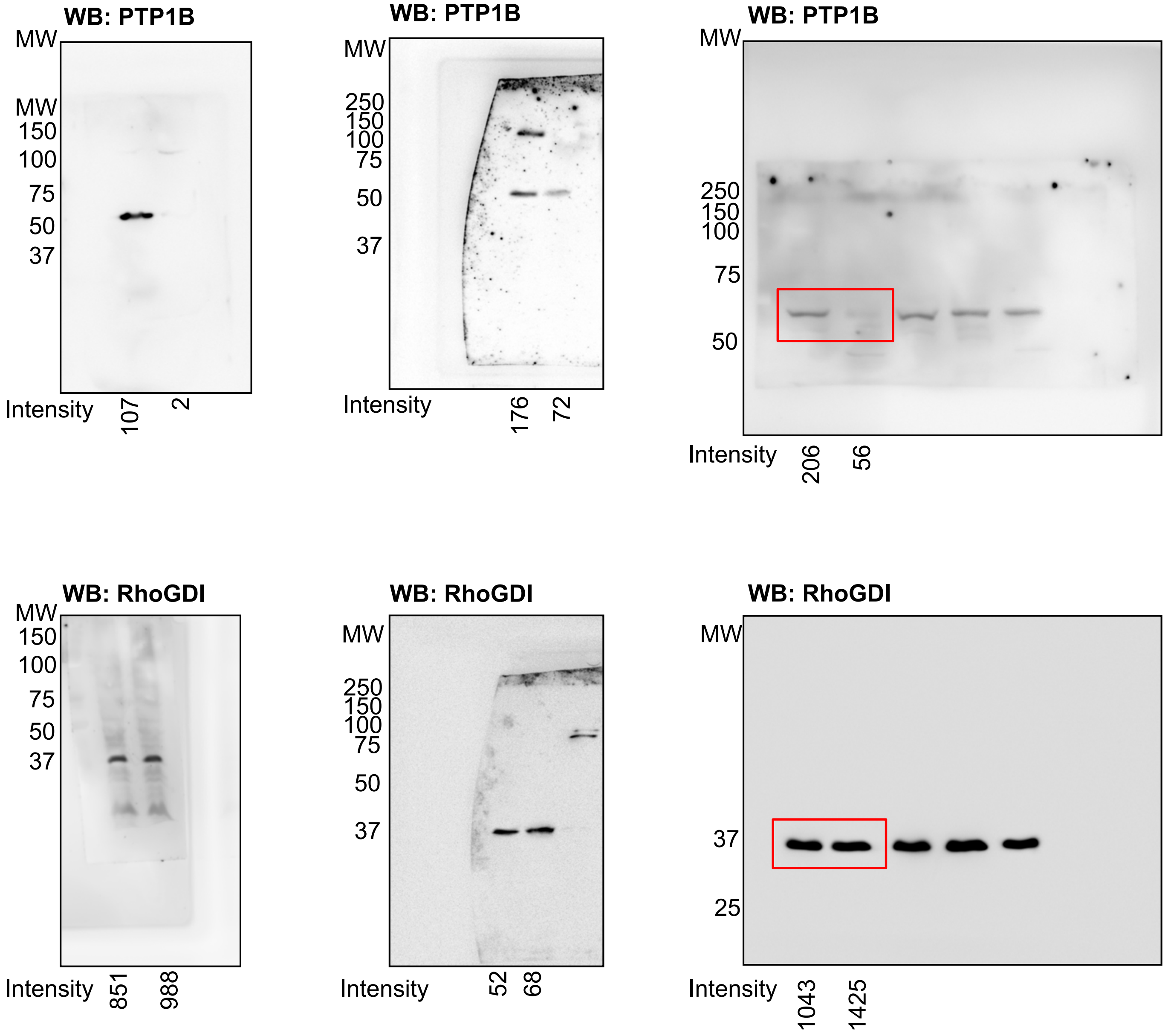

Figure 4B (left)

WB: pY530-Src

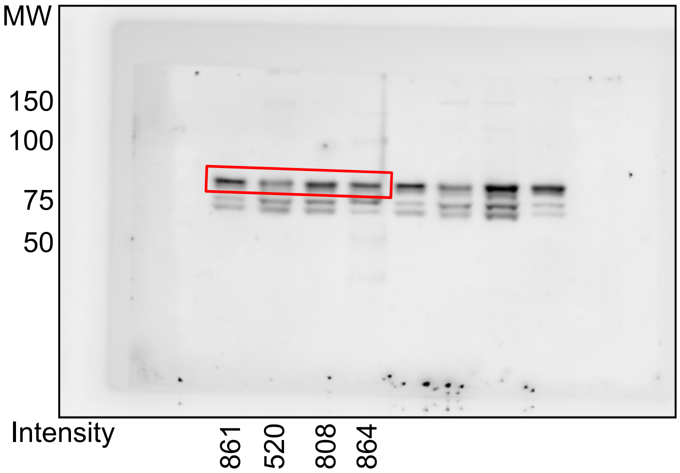

Figure 4B (center)

WB: pY530-Src

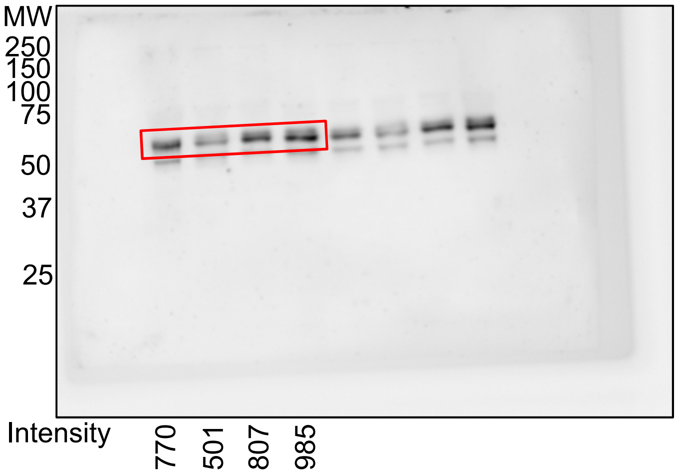

WB: Src

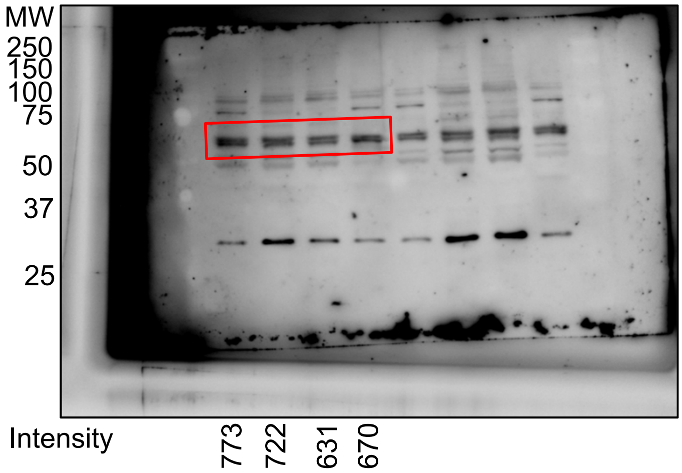

WB: Src

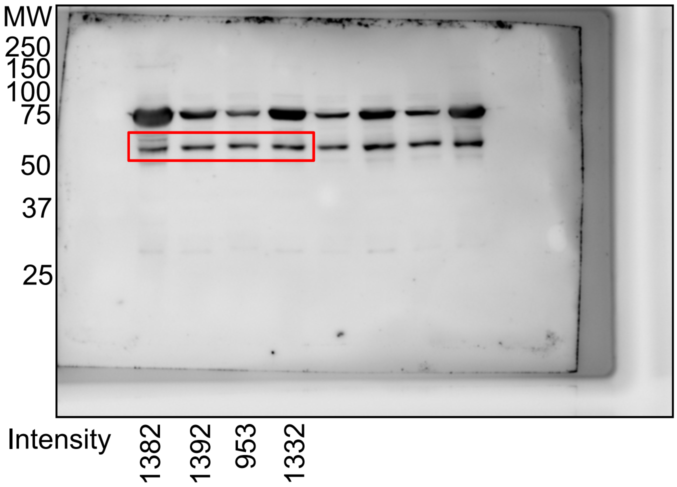

Figure 4B (left)

WB: pY530-Src

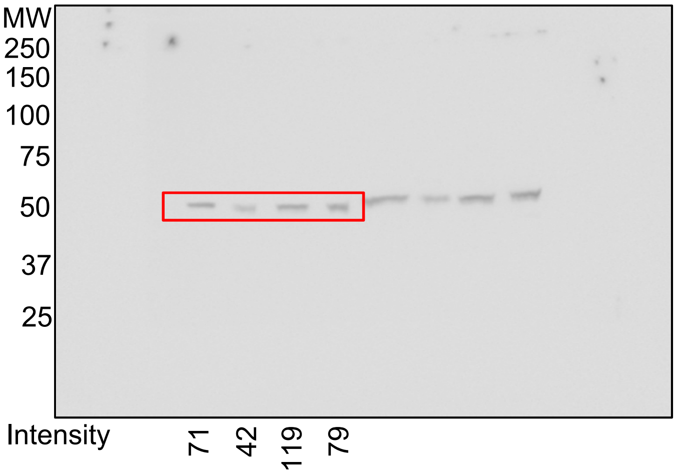

WB: Src

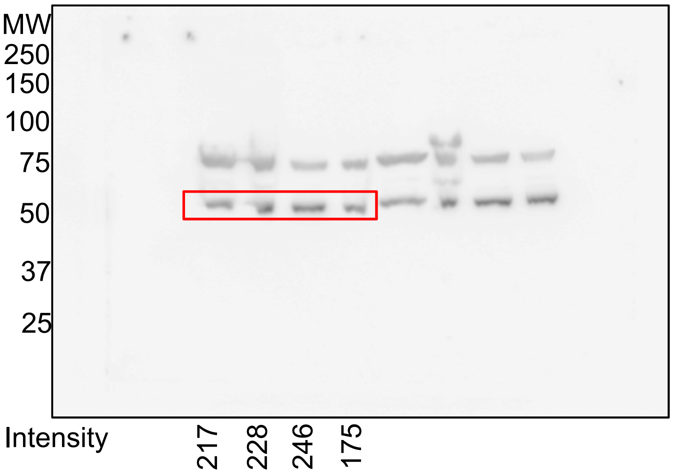

Figure 4C (left)

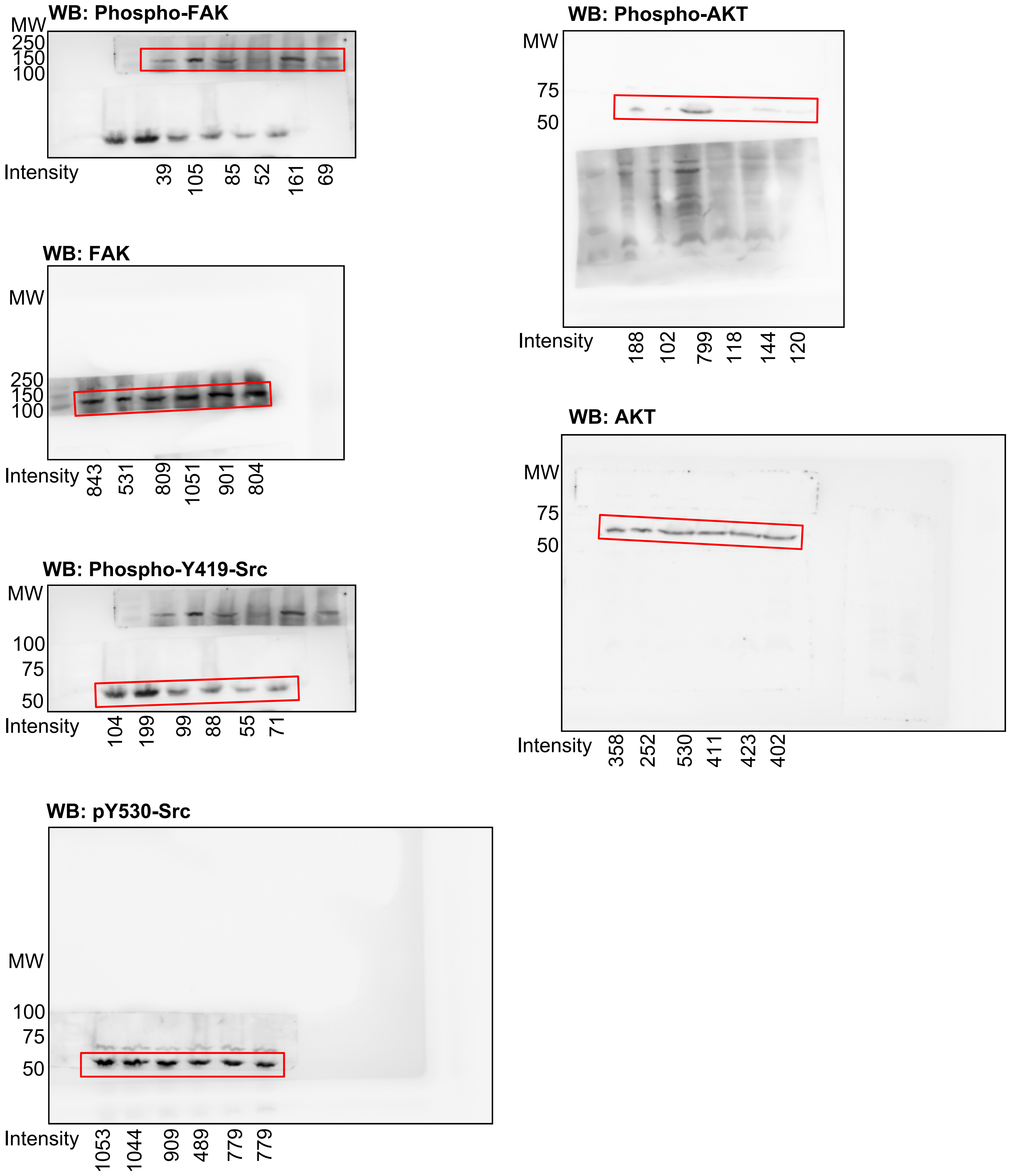

Figure 4C (left)

WB: Phospho-EKR1/2

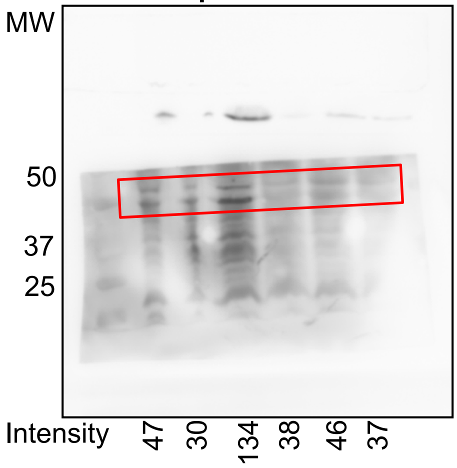

WB: ERK1/2

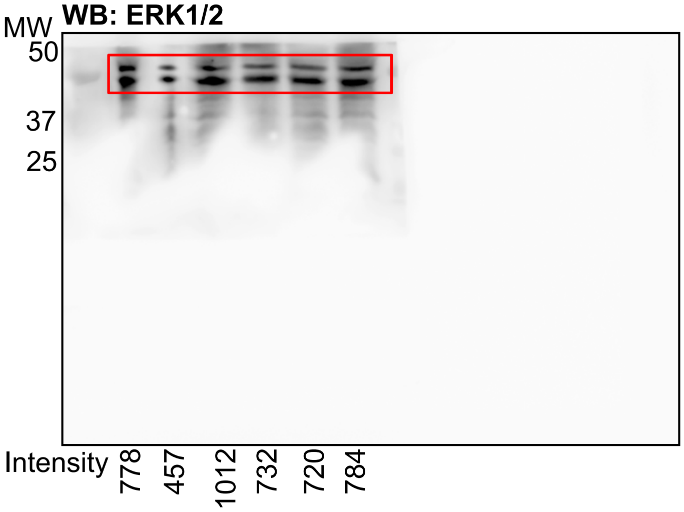

WB: RhoGDI

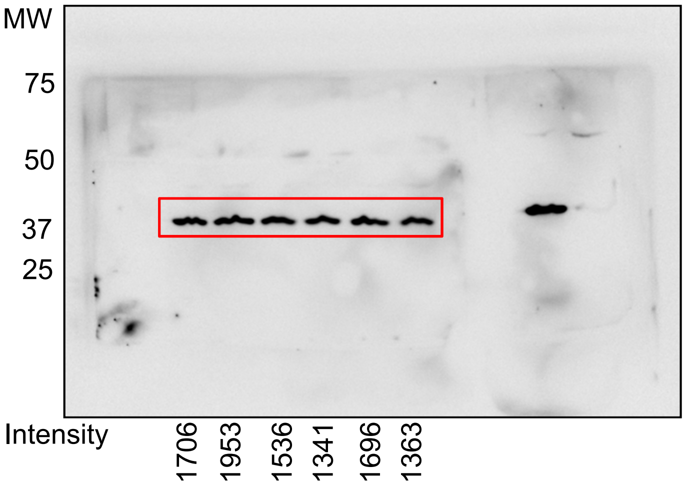

Figure 4C (right)

WB: Phospho-FAK

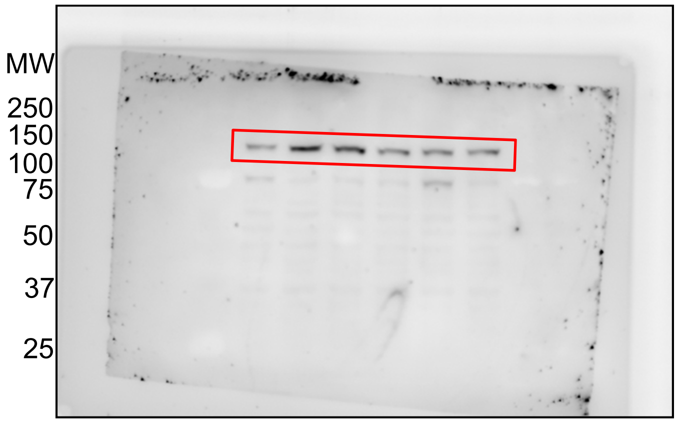

Intensity 80 253 221 143 133 136

WB: Src

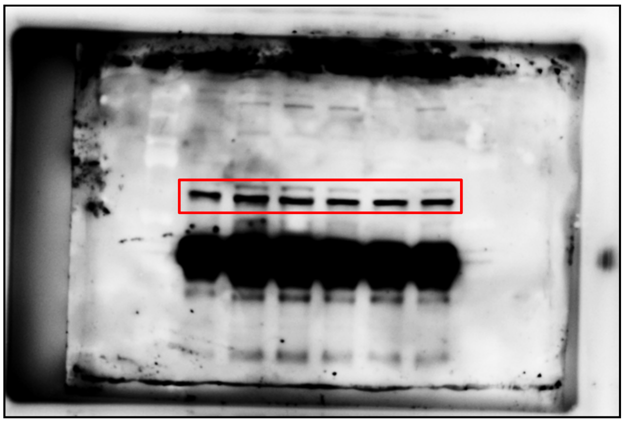

Intensity 94 155 93 104 109 93

WB: FAK

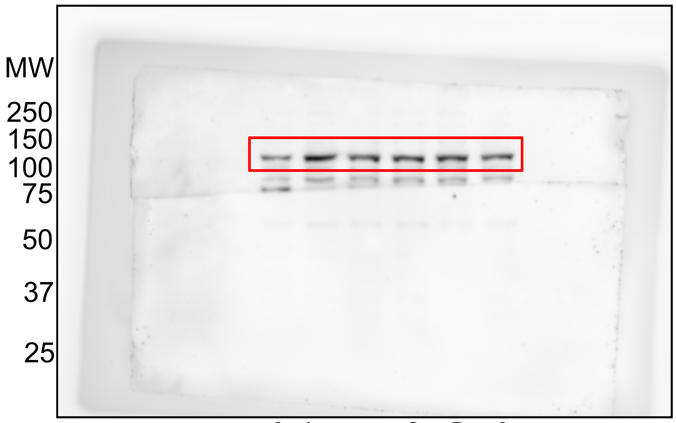

Intensity 318 707 451 535 629 518

WB: Phospho-AKT

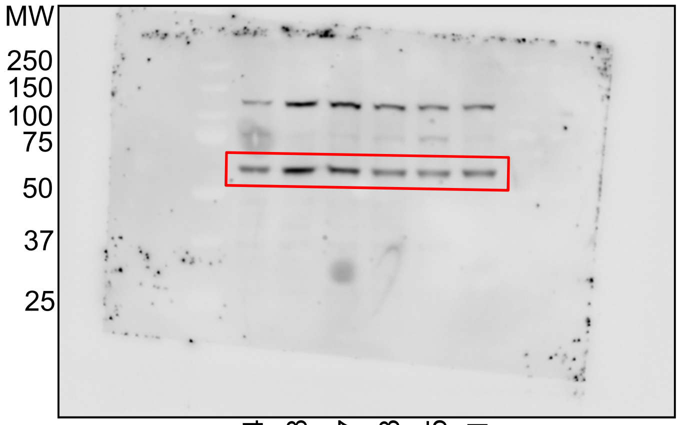

Intensity 34 68 67 43 35 41

WB: Phospho-Y419-Src

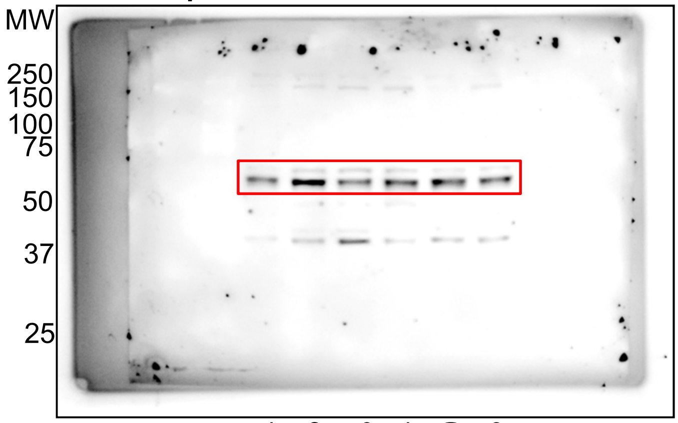

Intensity 94 155 93 104 109 93

WB: AKT

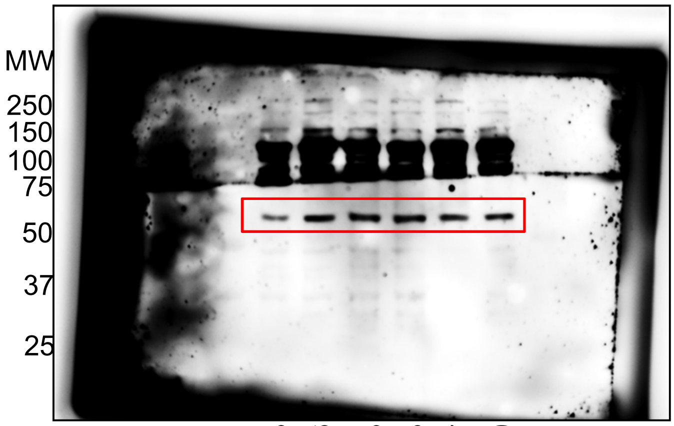

Intensity 48 66 63 63 57 60

Figure 4C (right)

WB: Phospho-EKR1/2

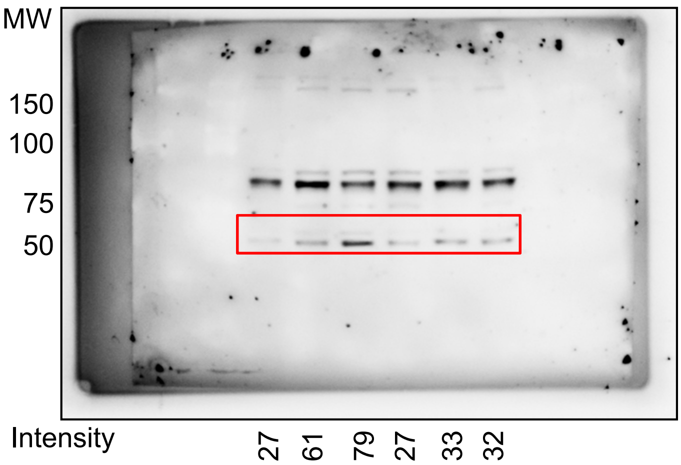

WB: ERK1/2

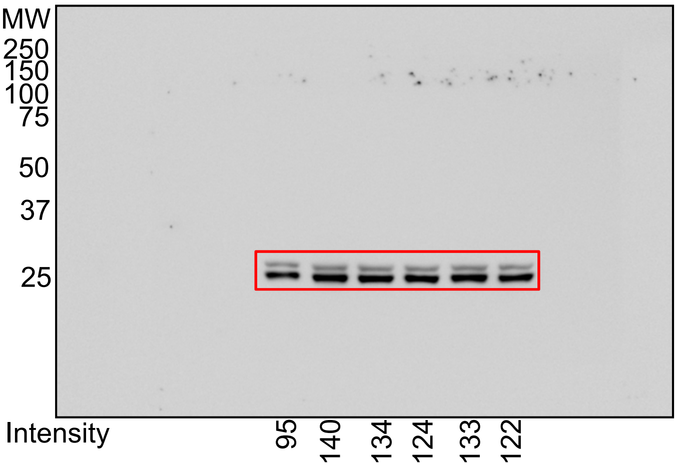

WB: RhoGDI

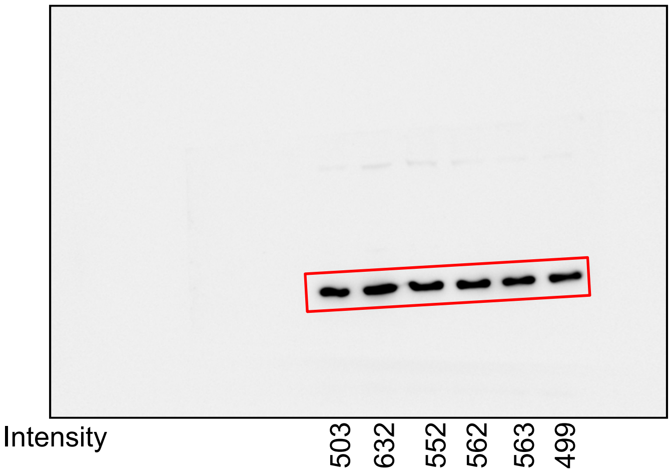

Figure 5A (up)

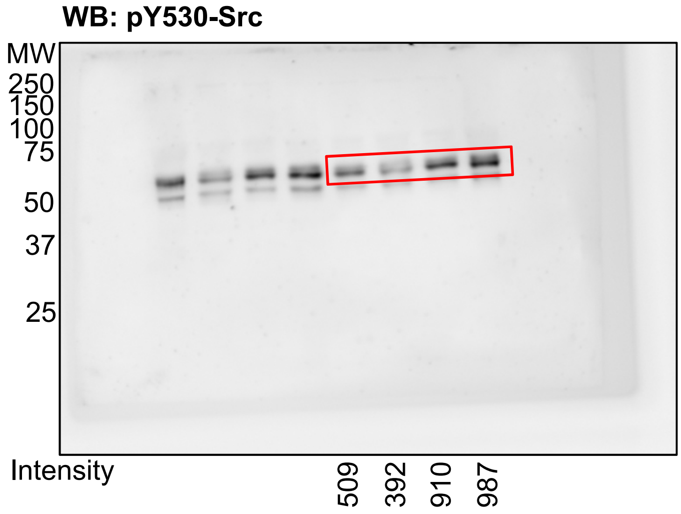

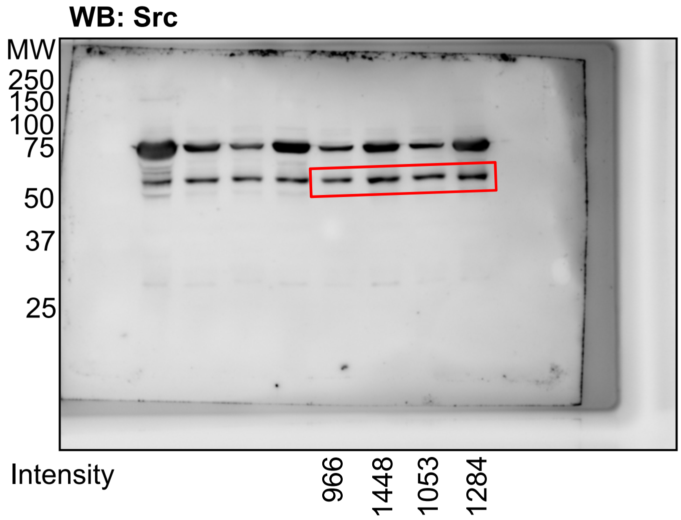

Figure 5A (center)

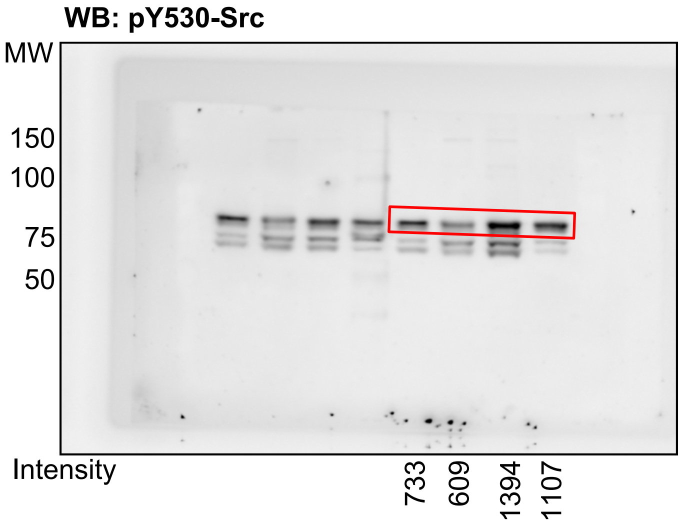

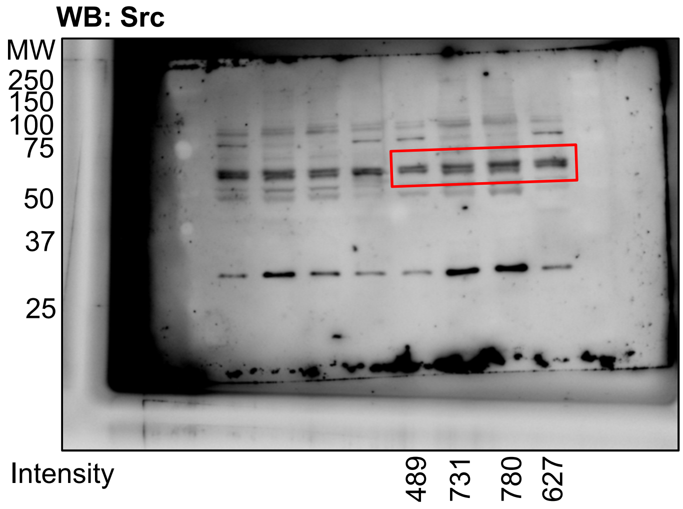

Figure 5A (down)

WB: pY530-Src

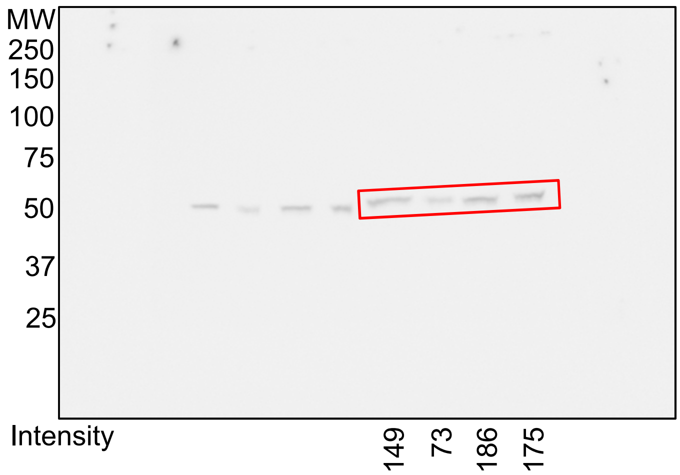

WB: Src

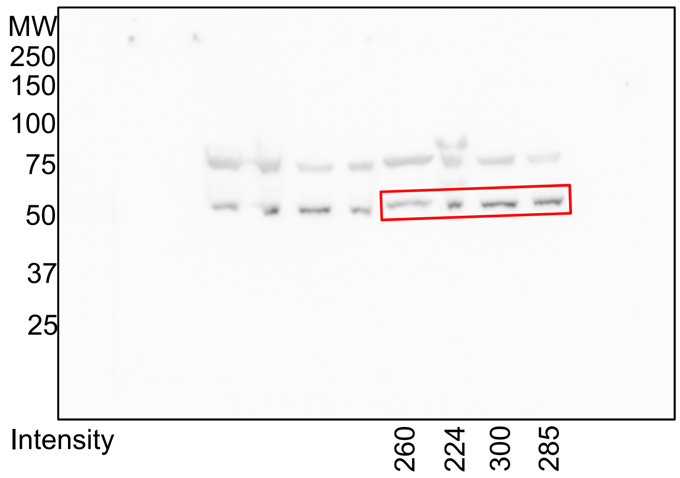

Figure 6A

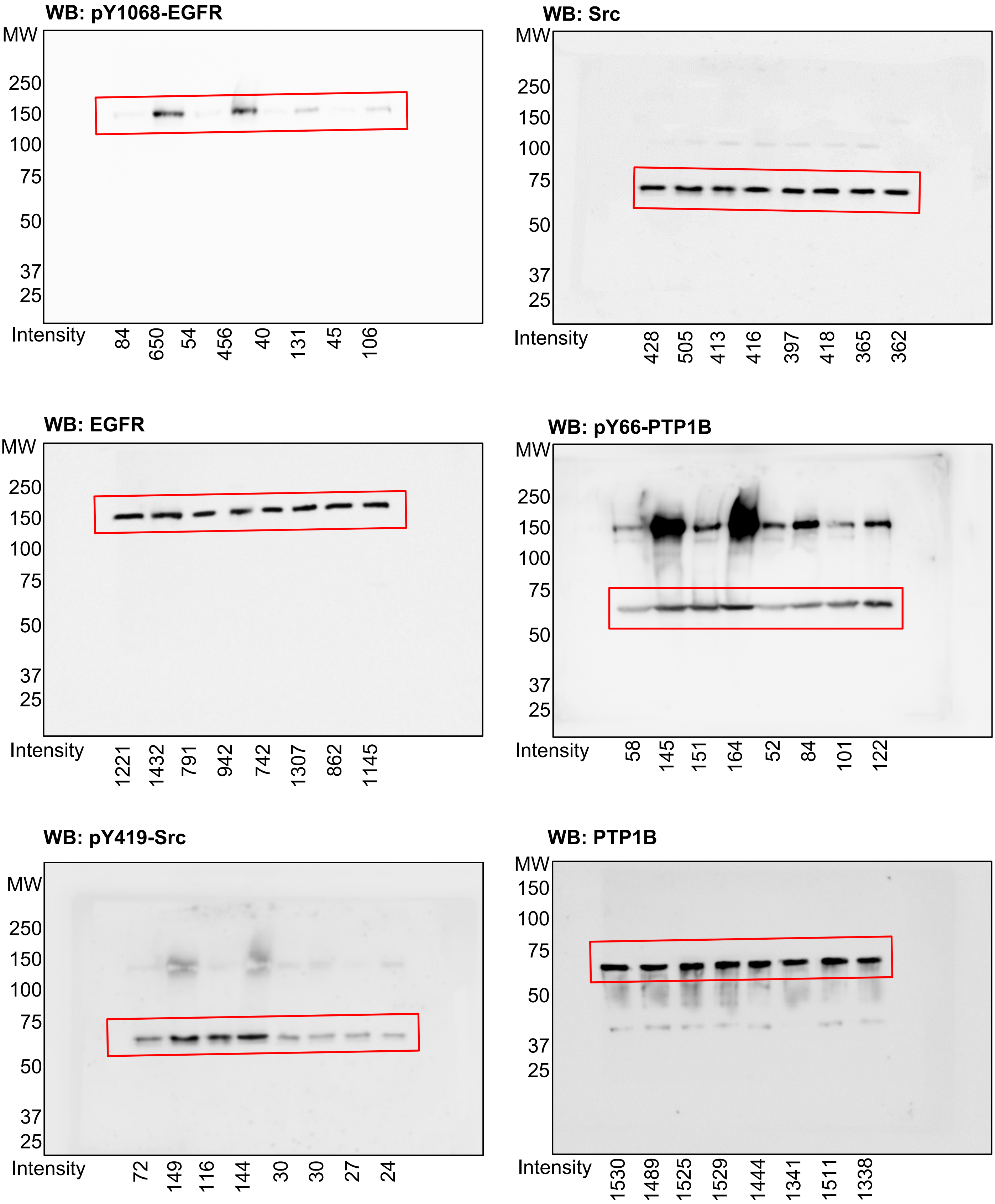

WB: pY1068-EGFR
MW
250
150
100
75
50
37
25
Intensity
84
650
54
456
40
131
45
106
WB: Src
MW
250
150
100
75
50
37
25
Intensity
428
505
413
416
397
418
365
362
WB: EGFR
MW
250
150
100
75
50
37
25
Intensity
1221
1432
791
942
742
1307
862
1145
WB: pY66-PTP1B
MW
250
150
100
75
50
37
25
Intensity
58
145
151
164
52
84
101
122
WB: pY419-Src
MW
250
150
100
75
50
37
25
Intensity
72
149
116
144
30
30
27
24
WB: PTP1B
MW
150
100
75
50
37
25
Intensity
1530
1489
1525
1529
1444
1341
1511
1338

Figure 6B (left)

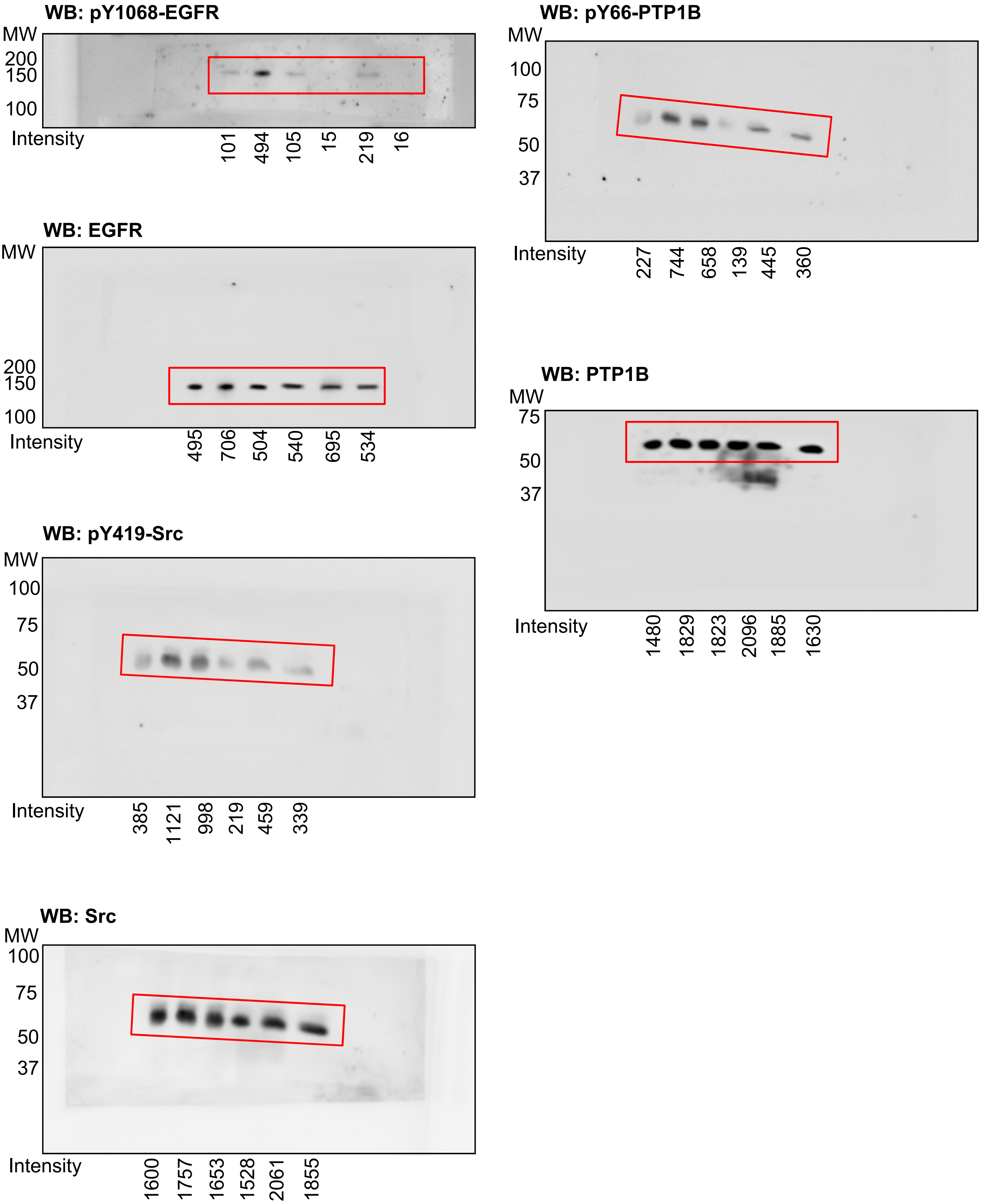

Figure 6B (center)

WB: pY1068-EGFR

MW
200
150
100

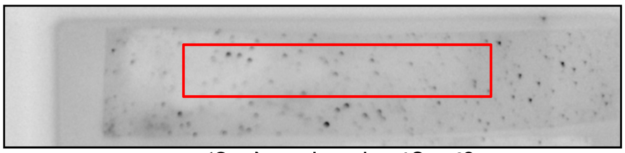

Intensity 6 7 4 4 5 8

WB: EGFR

MW
200
150
100

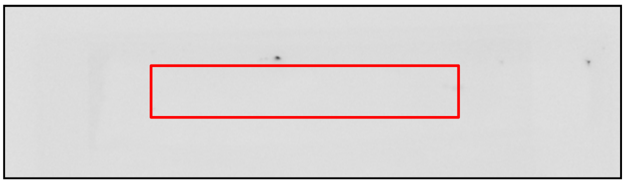

Intensity 6 10 4 5 4 5

WB: pY419-Src

MW
100
75
50
37

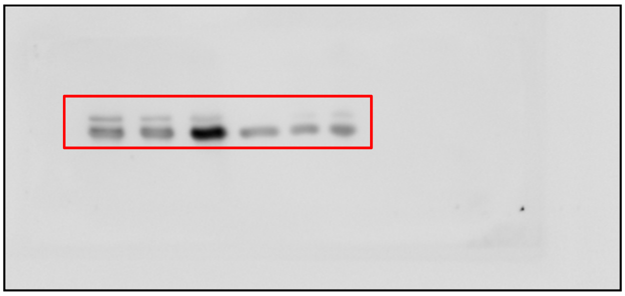

Intensity 814 673 1563 325 258 390

WB: Src

MW
100
75
50
37

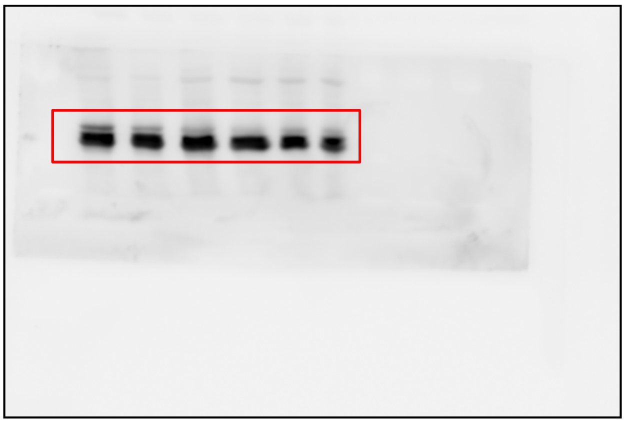

Intensity 2611 2235 2631 2388 1821 1571

WB: pY66-PTP1B

MW
100
75
50
37

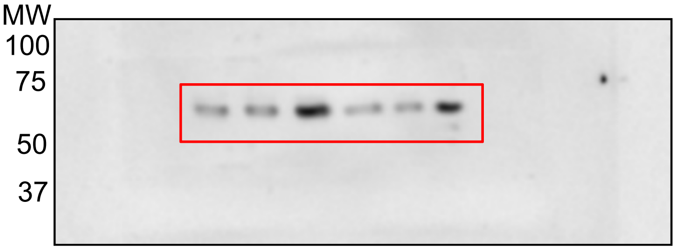

Intensity 528 571 1312 303 270 572

WB: PTP1B

MW
100
75
50
37

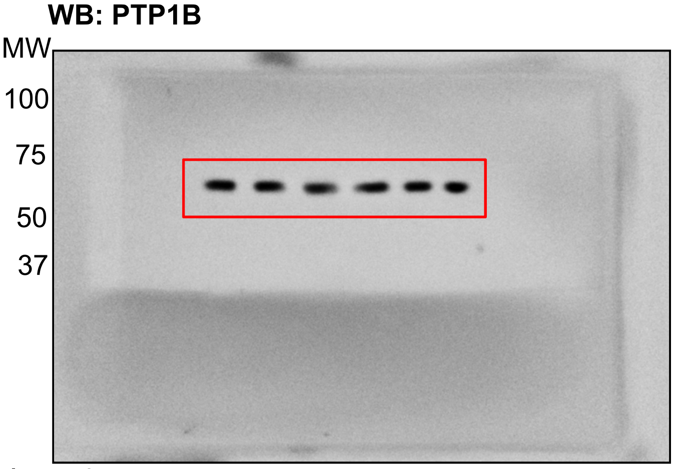

Intensity 1982 1971 2100 2221 1869 1599

Figure 6B (right)

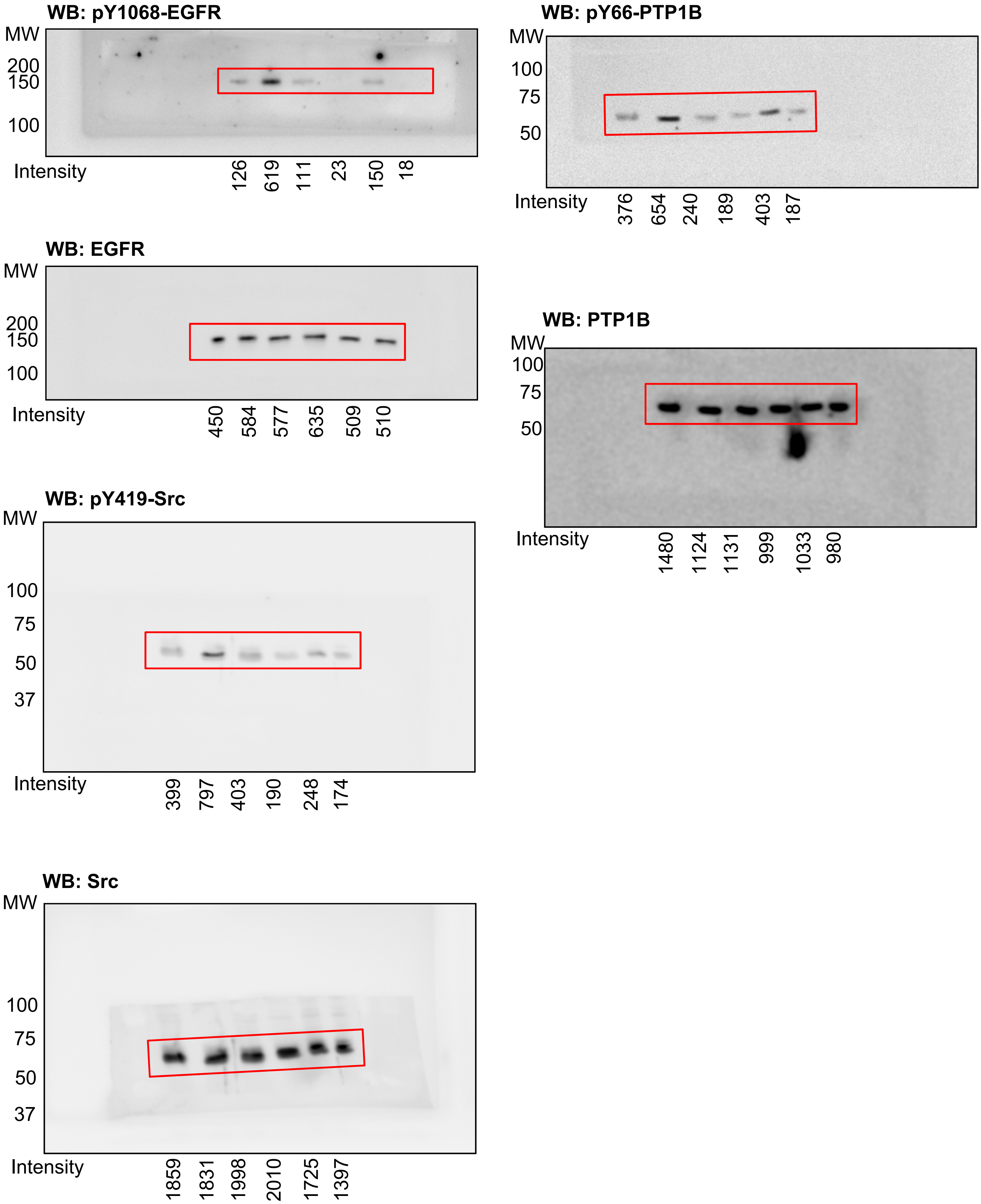

Supplement: Supplementary file 1 [file cancers-12-00500-s001.zip › suppl-final/western blot figures.pdf]
